# pHEMA Encapsulated PEDOT-PSS-CNT Microsphere Microelectrodes for Recording Single Unit Activity in the Brain

**DOI:** 10.3389/fnins.2016.00151

**Published:** 2016-04-18

**Authors:** Elisa Castagnola, Emma Maggiolini, Luca Ceseracciu, Francesca Ciarpella, Elena Zucchini, Sara De Faveri, Luciano Fadiga, Davide Ricci

**Affiliations:** ^1^Center for Translational Neurophysiology of Speech and Communication, Istituto Italiano di TecnologiaFerrara, Italy; ^2^Department of Smart Materials, Istituto Italiano di TecnologiaGenova, Italy; ^3^Section of Human Physiology, University of FerraraFerrara, Italy

**Keywords:** carbon nanotubes, PEDOT, pHEMA, microelectrode, neural recordings, biocompatibility

## Abstract

The long-term reliability of neural interfaces and stability of high-quality recordings are still unsolved issues in neuroscience research. High surface area PEDOT-PSS-CNT composites are able to greatly improve the performance of recording and stimulation for traditional intracortical metal microelectrodes by decreasing their impedance and increasing their charge transfer capability. This enhancement significantly reduces the size of the implantable device though preserving excellent electrical performances. On the other hand, the presence of nanomaterials often rises concerns regarding possible health hazards, especially when considering a clinical application of the devices. For this reason, we decided to explore the problem from a new perspective by designing and testing an innovative device based on nanostructured microspheres grown on a thin tether, integrating PEDOT-PSS-CNT nanocomposites with a soft synthetic permanent biocompatible hydrogel. The pHEMA hydrogel preserves the electrochemical performance and high quality recording ability of PEDOT-PSS-CNT coated devices, reduces the mechanical mismatch between soft brain tissue and stiff devices and also avoids direct contact between the neural tissue and the nanocomposite, by acting as a biocompatible protective barrier against potential nanomaterial detachment. Moreover, the spherical shape of the electrode together with the surface area increase provided by the nanocomposite deposited on it, maximize the electrical contact and may improve recording stability over time. These results have a good potential to contribute to fulfill the grand challenge of obtaining stable neural interfaces for long-term applications.

## Introduction

The stability of the interface between neural tissue and chronically implanted microelectrodes is crucial for recording and stimulation, both for research and clinical purposes, such as localization and prediction of epileptic seizures, control of pathological neural activity in Parkinson's disease, treatment of motor and sensory impairments (Xindong et al., 1999; Polikov et al., 2005; Hatsopoulos and Donoghue, 2009). Unfortunately, despite the strong efforts of researchers in this direction, a stable long-term bidirectional access to brain cells still remains a challenge.

Many events that occur when the electrode is inserted in the brain play a role in the failure of the implant. The main enemy of chronic implants is the gliosis, which is the final result of acute and chronic tissue reactions that take place around the implanted device. Micro-motions of the brain around the implanted electrode produce injuries that keep active the inflammatory response which leads to the subsequent formation of a glial scar (Agorelius et al., 2015). The presence of the scar tissue around the electrode impairs both signal recording and neural stimulation, displacing surrounding neurons and electrically insulating the electrode from the neighboring brain regions (Polikov et al., 2005; Marin and Fernández, 2010). Moreover, the insertion of the electrode into the brain triggers oxidative stress events at the device-tissue interface which directly impact on the survival of both electrode and neurons. Chemical redox reactions that occur on the surface of the electrode corrode the metal of the probe and the formation and release of oxygen reactive species from cell bodies are toxic causing neuron death (Potter-Baker and Capadona, 2015).

A variety of strategies for minimizing these adverse tissue reactions have been followed. Among them, the delivery of bioactive molecules during or after implant (Zhong and Bellamkonda, 2007; Jhaveri et al., 2009; Mercanzini et al., 2010; Yue et al., 2013), the electrode miniaturization in conjunction with the use of high surface area nanomaterial coatings that increase their recording and stimulation performances (Abidian and Martin, 2008; Kotov et al., 2009; Castagnola et al., 2010, 2013, 2014a; Castagnola V. et al., 2014; Kim et al., 2010; Kozai et al., 2012; Depan and Misra, 2014; Kolarcik et al., 2015), the use of hydrogel coatings or flexible substrates in order to reduce the stiffness mismatch between the implanted device and tissue (Polikov et al., 2005; Lind et al., 2010; Aregueta-Robles et al., 2014; Castagnola et al., 2014b; Castagnola V. et al., 2014; Agorelius et al., 2015; Khodagholy et al., 2015; Kozai et al., 2015).

In this work, we show that it is possible to combine several of these techniques for the development of a neural interface that may lead to an improved compatibility with the neural tissue. The starting point is a device based on a gold microsphere grown on the tip of a thin insulated platinum wire, coated with a nanostructured PEDOT-PSS-CNT composite (Castagnola et al., 2014b, 2015). This device already benefits from properties that can potentially minimize adverse tissue reactions: its spherical shape should help in keeping it in place reducing the compression forces exerted by the tissue, the thin tether between microprobe and external connection to the recording/stimulation system is able to accommodate relative movements of the brain with respect to the skull, and finally, the PEDOT-PSS-CNT coating ensures a very low impedance and high charge transfer capability. In this paper, we have added a further step encapsulating the microprobe with a soft synthetic permanent biocompatible hydrogel, Poly(2-hydroxyethyl methacrylate) (pHEMA), while fully preserving the properties of the nanostructured PEDOT-PSS-CNT electrode.

The pHEMA hydrogel encapsulation has the purpose to avoid a direct contact of nanomaterials with the brain tissue and to act as a physical barrier to their possible detachment. Furthermore, thanks to its softness, it helps in reducing the mechanical stiffness mismatch between electrode and cerebral tissue. Compared to fibrin hydrogel coatings previously used by our group (Castagnola et al., 2013; De Faveri et al., 2014) that are fully reabsorbed by the surrounding tissue within 14 days after the implant, the use of pHEMA allows to considerably increase the time span of its action. In this work, experiments have been performed on gold microspheres having diameters similar to those used in our previous work (Castagnola et al., 2015), but grown at the end of Pt wires with larger diameter (i.e., 50 μm) in order to facilitate manipulation during insertion. This work is indeed focused on the local reaction to various coatings and not on the rigidity of the conductor, a factor that may affect scar induction, that will require further studies, but that was here kept constant by always using the same type of wire. After preparation of the microprobes, we tested their ability to acquire high quality single unit recordings during both acute and chronic *in vivo* implants in the rat brain through signal to noise ratio evaluation. To monitor the evolution of the interface between electrode and tissue during chronic implants, a correlation between recording quality and *in vivo* electrochemical properties was carried out. Scanning electron microscopy imaging and energy dispersive spectroscopy of explanted microelectrodes were used to assess the stability of pHEMA coating after implants having a duration which varied from a few hours to 4 weeks.

## Materials and methods

### Microprobe realization and testing

#### Implantable gold microspheres

Microspheres of fuzzy gold are directly grown by electrochemical deposition at the end of a 50 μm diameter platinum core wire insulated by a 10 μm thick polyimide layer (Good Fellow, England), starting from a 10 mM potassium dicyanoaurate (I) (Aldrich Chemistry, Sigma Aldrich, USA) agar gel (0.1 wt%) (Fluka Biochemika, Spain), and applying monophasic voltage pulses (0.2–1.0 V, 240 s, duty cycle 50%) for 9 h using a potentiostat/galvanostat (PARSTAT 2273, Princeton Applied Research, USA). The temperature of the gel was kept at 45°C.

#### PEDOT-PSS-CNT electrochemical co-deposition

Poly(3,4-ethylenedioxythiophene)(PEDOT) and carboxylated MWCNTs (COOH-CNTs, NC 3151, < 4 % of COOH functional groups, Nanocyl S.A., Belgium) nanocomposites (PEDOT-CNT) were co-electrodeposited from a 0.5 M 3,4-ethylenedioxythiophene (EDOT, Sigma-Aldrich, USA) aqueous solution containing 1 mg/ml of suspended COOH-CNTs and 0.6 wt% of poly(sodium 4-styrenesulfonate)(PSS, Sigma-Aldrich, USA). COOH-CNTs were suspended in ultrapure water (Milli-Q, Millipore, USA) by horn sonication (Vibra-Cell VCX130, Sonics and Materials, USA) (6 s at 66% duty cycle pulses, 4 W/ml) for 30 min while keeping the solution cooled with an ice bath. PSS and the monomer were added to the suspension immediately afterwards, and the solution was kept deoxygenated by bubbling nitrogen. The electrochemical deposition was carried out in inert atmosphere in potentiostatic mode at constant temperature (ice bath, 0°C), using a polymerization potential of 0.8 V vs. reference electrode for 100 s. Depositions were carried out using a potentiostat/galvanostat (PARSTAT 2273, Princeton Applied Research, USA) connected to a three-electrode electrochemical cell with a platinum counter electrode and an Ag/AgCl reference electrode.

#### pHEMA hydrogel coating

Poly(2-hydroxyethyl methacrylate) (pHEMA) hydrogel was polymerized directly on the PEDOT-PSS-CNT coating after a single dipping into a prepolymer aqueous solution containing 62.5% (w/w) 2-hydroxyethyl methacrylate (HEMA), 0.003% (w/w) ethylene glycol dimethacrylate (EDGMA) and 0.04% (w/w) Irgacure 651 (all from Aldrich Chemistry, Sigma Aldrich, USA). Polymerization was obtained by exposure to UV light (365 nm, 350 μW/cm^2^) for 2 h (ENF-260C/FE, Spectroline, Spectronics Corporation, USA). For optical microscopy, the pHEMA precursor solution was stained using Ponceau Xylidine dye (Chroma, Germany).

#### Optical and surface characterization of coated microelectrodes

Gold, PEDOT-PSS-CNT and pHEMA coatings where routinely examined via optical microscopy using a Leica Zoom APO 16 equipped with a Leica DFC290 digital camera (Leica Microsystems, Germany). Morphology prior and after brain implant was studied through scanning electron microscopy (SEM) using a JEOL JSM-6490LA SEM (JEOL, Japan) and a Zeiss EVO 40 SEM (Zeiss, Germany). High resolution imaging of PEDOT-PSS-CNT coated devices was performed using a Jeol JSM-7500FA FEG SEM. Surface composition of devices was assessed by energy dispersive spectroscopy (EDS) using a INCA 300 (Oxford Instruments, England) mounted on the Zeiss EVO 40 SEM.

#### Electrochemical characterization

The electrochemical behavior of the microelectrodes was studied in a 0.9% NaCl aqueous solution, by cyclic voltammetry (CV) to quantify their capacitive charging, and by electrochemical impedance spectroscopy (EIS) to determine the electrical properties of the system over a large range of frequencies. During the CV tests, the working electrode potential was swept between 0.6 and −1 V vs. Ag/AgCl, maintaining a scan rate of 100 mV/s. During the EIS measurements, a sine wave (10 mV RMS amplitude) was superimposed onto the open circuit potential while varying the frequency from 1 to 10^5^ Hz. EIS and CV were carried out using a potentiostat/galvanostat (Reference 600, Gamry Instruments, USA) connected to a three-electrode electrochemical cell with a platinum counter electrode and a Ag/AgCl reference electrode.

#### Mechanical measurements

The mechanical response of the pHEMA hydrogel was characterized through uniaxial compression tests on 10 mm diameter, 5 mm thick samples. Tests were performed on a dual column universal testing machine (Instron 3365, USA) under a displacement rate of 1 mm/min. The Young's modulus *E* was calculated from the initial linear slope of the stress-strain curve. Seven repetitions were performed.

### *In vivo* testing and histology

#### Animals

Seventeen adult male Wistar rats weighing 250–270 g were used: six for acute recordings and eleven for chronic experiments. Details are reported in Table 1. All experimental subjects were bred in the breeding facility of the University of Ferrara. The experiments were carried out in accordance with the guidelines established by the European Communities Council (Directive 2010/63/EU of September 22nd, 2010) and the protocol was approved by the Italian Ministry of Health (Legislative decree n. 26 of March 2014), authorization n° 332/2015-PR.

**Table 1:** **Number of animals and probes used during acute experiments and chronic implants**.

**Acute experiments**				
	**Animals**	**Recording positions**	**PEDOT-PSS-CNT**	**pHEMA-encapsulated**
	6	20	6	6
**Chronic implants**				
**Duration**	**Animals**	**Hemispheres**	**PEDOT-PSS-CNT**	**pHEMA-encapsulated**
14 days	8	16	16	16
28 days	3	4	6	6

*It is specified the number of recording positions for acute experiments and the total number of hemispheres implanted in chronic experiments*.

#### Animal surgery for signal recording

Wistar rats were anesthetized with a mixture of Zoletil (Virbac, France; 30 mg/kg) and Xylazine (Bayer, Germany; 5 mg/kg) administered intraperitoneally (i.p.). For the duration of the whole procedure, the depth of anesthesia was monitored by testing the absence of hind limb withdrawal reflex and was maintained by additional i.m. doses of anesthetic. The body temperature was maintained at 37–38°C with a thermostatically controlled heating pad and lacrigel (Farmigea, Italy) was placed on eyes to avoid dryness. After shaving and swabbing the head with ethanol, the anesthetized animal was placed in a stereotaxic apparatus (David Kopf Instruments, USA) equipped with Ear Bars (Model 957 for small animals). An approximately 2 cm long incision was made along the midline of the cranium. The underlying muscle and connective tissue were retracted to expose the skull. A craniotomy (5 × 5 mm) was made in the parietal bone to expose the somatosensory cortex identified according to vascular landmarks and stereotaxic coordinates (Hall and Lindholm, 1974; Chapin and Lin, 1984; Paxinos and Watson, 2007). Sterile saline solution was applied while drilling to avoid any local heating and to keep clean the bone surface.

The exposed dura mater was wetted with saline and carefully incised using surgical micro-scissors and the tip of a 24 G syringe needle to producing an opening both in the dura and in the pia mater. For acute recording sessions, microsphere electrodes were lowered into the cortex and 35 min of the spontaneous activity was recorded. For each rat, several traces for the two types of microelectrodes (PEDOT-PSS-CNT microspheres and pHEMA-encapsulated PEDOT-PSS-CNT microspheres) were recorded from different points of the cortex, at antero-posterior coordinates comprised between −1.5 and −4.5 mm and medio-lateral coordinates comprised between 2.5 and 4.5 mm. At the end of the recording sessions, the placement of electrodes was confirmed by histological section.

#### Microelectrode chronic implantation

For the chronic recordings, a custom-made support holding two microsphere microelectrodes spaced 1 mm apart was used. For each experiment, one PEDOT-PSS-CNT microsphere and one pHEMA encapsulated PEDOT-PSS-CNT microsphere were implanted in the parietal cortex of both hemispheres. The dura and pia mater were incised with a needle and the microelectrodes were manually advanced into the cortex. After implant, the surface of the implanted tissue was protected using Kwik-Sil silicone polymer (World Precision Instruments Inc, USA) and both supports, excluding the connector, were cemented to the skull using dental acrylic (Jet Repair Acrylic; Lang Dental Manufacturing, USA). To hold in place the dental acrylic, four stainless steel bone screws were inserted into the skull and a stainless steel ground wire was attached to the nearest screw as a reference. The skin was sutured around the cement, gentamycin cream (Mylan s.p.a., Italy) was spread over the wound and finally an antibiotic solution of Baytril 5% was administered (Bayer, Germany, 0.5 ml/10 kg, i.m.).

#### Neural recordings

Neural recordings from the somatosensory cortex were performed to characterize the electrical performance of the microelectrodes *in vivo*. Electrophysiological data were acquired using TDT RZ-2 Processor and PZ2 preamplifier (Tucker-Davis Technologies, USA). The gain was set to 1. The acquired neural traces were sampled at 24,414 Hz and bandpass filtered from 10 to 5000 Hz. To connect the microelectrodes to the headstage, a custom support for ZIF-Clip (Tucker-Davis Technologies, USA) was designed. Recorded data were stored and analyzed off-line using the Off-Line Sorter software (Plexon Inc, USA). In chronic recordings, data were acquired at day 1, 7, 14, 21, and 28 after the implant.

#### Histology and immunofluorescence

At the end of the recording sessions, the animals were maintained deeply anesthetized and transcardially perfused with 300 ml of 0.9% saline solution at room temperature followed by 500 ml cold fixative solution of 2.0% paraformaldehyde, 1.25% glutaraldehyde and 2.0% sucrose (all from VWR, USA), prepared in 500 ml of 0.1 M sodium phosphate buffered solution (PBS, pH, 7.4). Brains were then removed, postfixed overnight at 4°C and placed in a 30% sucrose-buffered solution until they sank. They were then frozen and 50 μm-thick coronal sections were cut using a sliding microtome (SM2000R; Leica Microsystems, Canada).

To determine the (localization) layer of the recording, the sections belonging to the rats of acute session were stained with thionin (Sigma Aldrich, USA) and viewed under brightfield illumination with an Olympus BX51 microscope (Olympus, USA) coupled with a color video camera CX-9000 (MicroBrightField, USA) and with the NeuroLucida system (MicroBrightField, USA). Thionin-stained sections containing the electrode traces were acquired at 125 × magnification (see Supplementary Figure 6S).

In order to investigate the tissue response to electrode implant the immunofluorescence staining was performed at 2 weeks (1 rat, 2 hemispheres, 2 PEDOT-PSS-CNT and 2 pHEMA-encapsulated microspheres) and 4 weeks (2 rats, 3 hemispheres 4 PEDOT-PSS-CNT and 4 pHEMA-encapsulated microspheres) after implant. The brain sections were stained using antibodies directed against reactive astrocytes detected by the production of glial fibrillary acidic protein (GFAP); activated microglia/macrophages detected by the membrane-bound CD68-antigen (clone-ED1); neuronal nuclei (NeuN) and total number of cell nuclei (DAPI), to label the principal cell lines involved in the inflammatory tissue reaction.

The adjacent sections were divided into two series, treated with blocking solution consisting of 4% (v/v) normal goat serum (Sigma Aldrich, USA), 0.5% (v/v) Triton-X-100 (Sigma Aldrich, USA), 2% (w/v) bovine serum albumin (BSA) (Sigma Aldrich, USA) in PBS for 1 h and then incubated in the primary antibodies overnight at room temperature. The first series was stained using mouse-anti-GFAP (1:500, Sigma Aldrich, USA) and rabbit-anti-NeuN (1:200, Millipore, USA) while the second one using mouse-anti-ED1 (1:300, Millipore, USA). After 3 rinses in PBS (10 min per rinse) the sections were incubated with the antirabbit-Alexa-488 and antimouse-Alexa-633conjugated secondary antibodies (1:500, Thermo Fisher Scientific, USA) for 4 h in the dark, at room temperature. All mentioned antibodies were used diluted in the blocking solution. Finally, after washing 3 times in PBS, the two series sections were mounted separately onto microscope slides, counterstained with ProLong® Gold Antifade Mountant containing DAPI (Thermo Fisher Scientific, USA) and covered with a coverglass. For the different antibody protocols, controls by omission of primary antibodies were negative.

The staining was observed using an BX51 microscope with 10×, 20×, 40× objectives (Olympus, Japan) and equipped with a X-Cite® 120 fluorescence microscopy illumination system (EXFO, Canada) and a color video camera CX-9000 (MicroBrightField, USA). The images of the fluorescence of the ED1-positive cells (red), GFAP-positive cells (red), neuron nuclei (green) and cell nuclei (blue) were acquired and analyzed using NeuroLucida (MicroBrightField, USA) and ImageJ software (developed at the National Institutes of Health, USA).

#### *In vivo* impedance measurements

The *in vivo* impedances of chronic implanted microspheres were analyzed through electrochemical impedance spectroscopy (EIS) using the two-electrode configuration. The implanted microspheres were referenced to a low impedance stainless steel bone screw inserted into the skull (see Section Microelectrode Chronic Implantation). The two-electrode method is suitable for measuring impedance from microelectrodes due to the large difference in impedance relative to the reference and the small current that passes through the circuit (Brett and Brett, 1993; Williams et al., 2007). During the EIS measurements, a sine wave (10 mV RMS amplitude) was imposed onto the open circuit potential while varying the frequency from 1 to 10^5^ Hz. EIS were carried out using a potentiostat/galvanostat (Reference 600, Gamry Instruments, USA). Impedance spectra measurements were repeated three times for each microelectrode at day 1, 7, 14, 21, and 28 after the implant.

#### Evaluation of signal-to-noise ratio

To evaluate the signal-to-noise ratio (SNR), each acquired trace was digitally high-pass filtered above 300 Hz (Butterworth, 4-poles). The signal was wavelet decomposed and thresholded to 4.5 standard deviations (SD) above and below the mean of the sample distribution to discriminate signal from noise. Waveforms were clustered using T-Distribution E-M Algorithm. This algorithm is a variant of the E-M algorithm by Shoham et al. (Figueiredo and Jain, 2003; Shoham et al., 2003), fits the available data points by adopting T distributions and provides a robust alternative to the use of gaussian mixture models, automatically down-weighting the effect of outlier waveforms. It starts by processing a large number of clusters and combines them together as the algorithm proceeds, so as to minimize a penalized likelihood function (Shoham et al., 2003). All the spikes with inter-spike-interval (ISI) smaller than the refractory period (2 ms) have been removed.

In the present article the method adopted to evaluate the SNR is based on computing the median of the distribution of the absolute value of the signal. The method is introduced by Dolan et al. (Dolan et al., 2009). Briefly, for Gaussian noise, the median of the absolute value of the signal will be 0.6745σ_*n*_, where σ_*n*_ is the standard deviation of the Gaussian noise. Therefore, the standard deviation of the noise can be estimated as follows:

(1) $$\begin{array}{l} \sigma_{n}=median\frac{\left|x\left(t\right)\right|}{0.6745} \end{array}$$

where *x(t)* is the original recorded signal.

Finally, the SNR was calculated as follows:

(2) $$\begin{array}{l} SNR=\frac{S}{\left(2\sigma_{n}\right)} \end{array}$$

where *S* is the signal amplitude defined as the peak-to-peak amplitude of the mean waveform for each cluster and the noise was defined as two times the estimated standard deviation of the noise.

The statistical significance between the average SNR, calculated for both type of microspheres, was assessed by a one-way ANOVA (*p* < 0.05 significance level). Values were expressed as mean ± Standard Error of Mean (SEM).

#### Signal power calculation

The signal was estimated by computing the spectral power densities (SPDs, data sampling rate 24,414 Hz, segmentation length of 512, zero-overlapping ratio of the segments, 2–5000 Hz window function) of unfiltered spike activities recorded using PEDOT-PSS-CNT and pHEMA-encapsulated microspheres. The signal spectral power over the spike frequency range was computed as the integral of the SPDs of the signals between 250 and 3000 Hz, the frequency range were individual spikes can be detected.

## Results

### Electrochemical and physical properties of microprobes

#### Electrochemical properties of PEDOT-PSS-CNT coated microspheres

**Figure 1:**
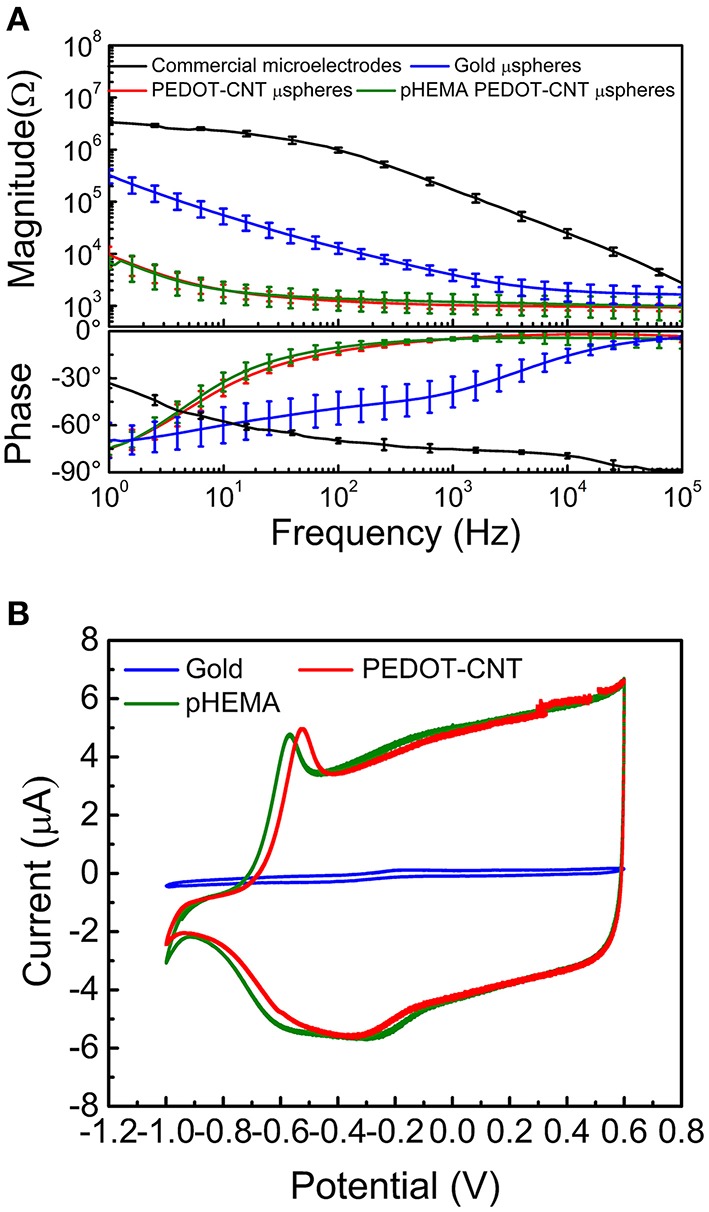
**(A)** Impedance spectra of commercial microelectrodes (black), gold microspheres before (blue) and after (red) PEDOT-PSS-CNT electrodeposition and (green) PEDOT-PSS-CNT coated microspheres after pHEMA encapsulation. **(B)** Sample cyclic voltammograms of a gold microsphere (blue) and a PEDOT-PSS-CNT coated microsphere before (green) and after (red) pHEMA encapsulation.

Coating the gold microspheres, that had a diameter in the range of 100 μm (106.5 ± 9.3 μm, *N* = 10) with PEDOT-PSS-CNT significantly reduces their impedance, as shown by the impedance spectra reported in Figure 1A. The impedance values of pristine gold microspheres are 3.96 ± 0.95 kΩ at 1 kHz, 13.04 ± 3.07 kΩ at 100 Hz and 324.04 ± 100.37 kΩ at 1 Hz (mean ± standard deviation, 10 samples), much lower than commercially available intracortical microelectrodes, i.e., quartz insulated platinum/tungsten tips (Ansaldo et al., 2011). PEDOT-PSS-CNT coating further decreases the impedance in all the frequency range, and especially in the low frequency band (1.04 ± 0.17 kΩ at 1 kHz, 1.24 ± 0.24 at 100 Hz and 9.56 ± 4.13 at 1 Hz). A second effect of PEDOT-PSS-CNT coating is the large increase in charge transfer capability (CTC), calculated as the time integral of an entire CV cycle between 0.6 and −1V, that passes from 101.6 ± 64.4 mC/cm^2^ of the pristine gold microspheres to 540.7 ± 70.3 mC/cm^2^ of the PEDOT-PSS-CNT coated ones. An example of CVs of pristine and PEDOT-PSS-CNT coated microspheres is shown in Figure 1B.

**Figure 2:**
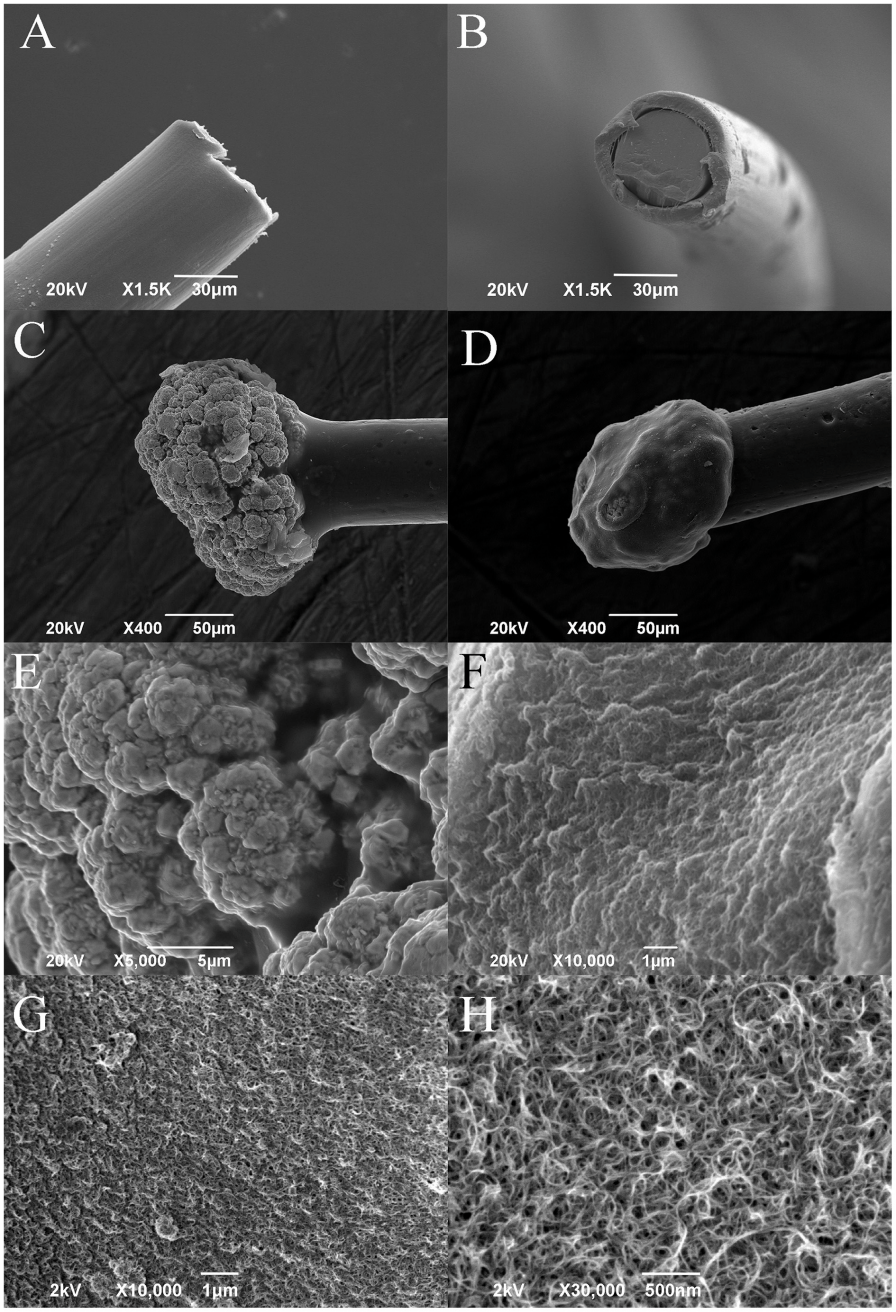
**Representative SEM images of (A,B) platinum wire before deposition (lateral and frontal view), (C) gold microsphere, and (D) PEDOT-PSS-CNT coated microsphere**. Higher magnification images of the surface morphology of **(E)** nanostructured gold and of **(F)** PEDOT-PSS-CNT composite obtained by Zeiss EVO 40 SEM. High resolution images of **(G,H)** PEDOT-PSS-CNT composite obtained by Jeol JSM-7500FA FEG-SEM.

SEM images of a platinum wire at beginning of the process (Figures 2A,B) after microsphere growth (Figure 2C,E) and after PEDOT-PSS-CNT coating (Figure 2D) are reported. Higher resolution imaging of PEDOT-PSS-CNT coated microspheres obtained with scanning electron microscopes having different performance, (Figure 2F) and (Figures 2G,H), shows the emergence of the fine nanoscale CNT scaffold structure, similar to what already demonstrated on flat electrode surfaces (Castagnola et al., 2013).

#### Electrochemical properties after pHEMA encapsulation of PEDOT-PSS-CNT microspheres

**Figure 3:**
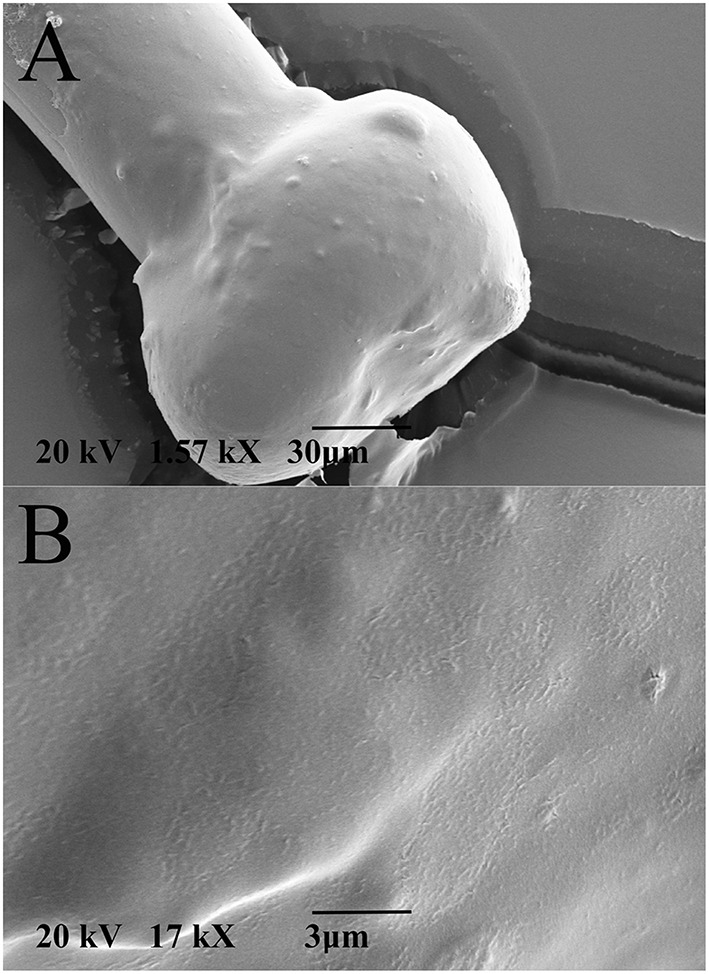
**Representative SEM micrographs of (A) pHEMA-encapsulated PEDOT-PSS-CNT microsphere and (B) higher magnification of the pHEMA coating**.

Two fundamental reasons lead us to encapsulate the PEDOT-PSS-CNT microspheres with pHEMA hydrogel. The first one was to enable a safe use of nanomaterials otherwise in direct contact with brain tissues (aiming at their use on human subjects) and the second was to reduce the mechanical mismatch in stiffness between electrode and cerebral tissue. As shown in Figure 3A, the pHEMA hydrogel evenly coats and encapsulates the microsphere and supporting wire, as also confirmed by optical imaging of dyed pHEMA (Supplementary Figure 1S). The pHEMA encapsulation preserves the electrochemical performance of PEDOT-PSS-CNT microspheres, as impedance and CTC values were maintained within the standard deviation value of non-encapsulated microprobes (1.2 ± 0.5 kΩ at 1 kHz, Figure 1A). Finally, we found that the pHEMA-encapsulated microelectrodes withstand the sterilization process in the absence of significant changes in impedance values (0.9 ± 0.6 kΩ @ 1 kHz before and 1.2 ± 0.9 kΩ @ 1 kHz after sterilization).

#### Sturdiness and electrochemical stability of pHEMA encapsulation

**Figure 4:**
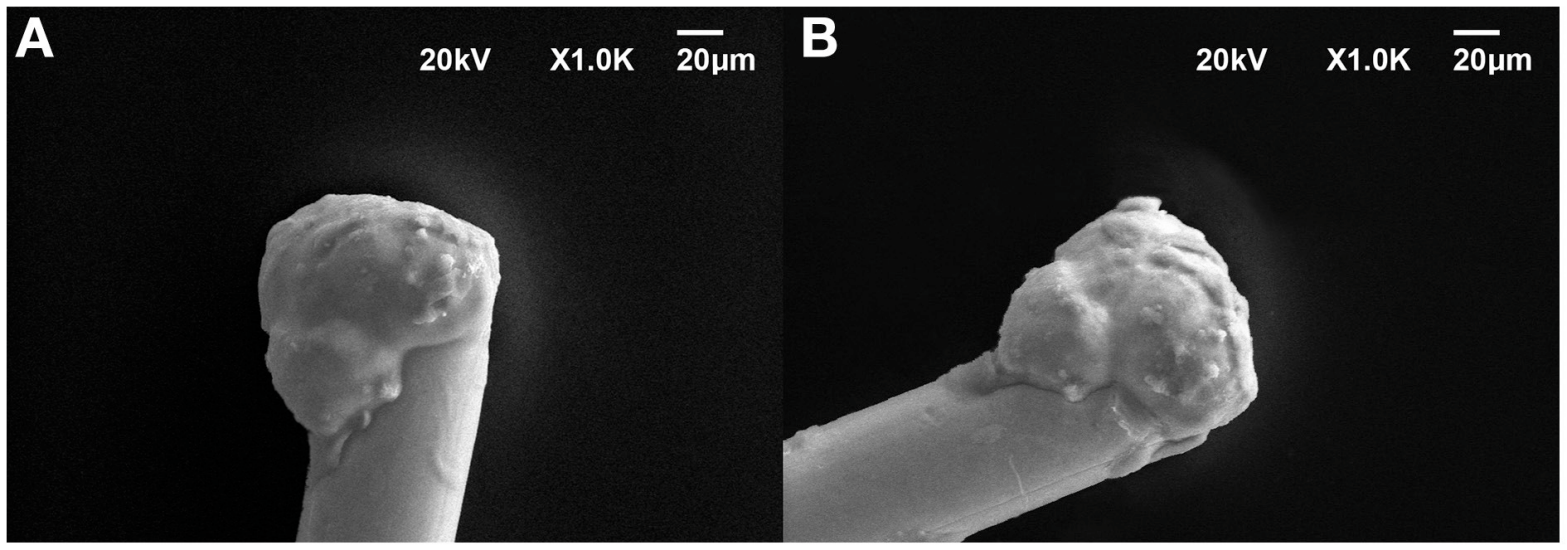
**Representative SEM micrographs of the same pHEMA-encapsulated PEDOT-PSS-CNT microsphere (A) before and (B) after one week immersion in a PBS solution containing 30 mM of H_2_O_2_ to mimick the situation where the device is implanted in the human body and hydrogen peroxide is generated by an inflammatory reaction**.

To perform *in-vitro* tests for evaluating the stability of pHEMA encapsulation, we have immersed pHEMA coated microspheres in a phosphate buffered saline (PBS) solution containing 30 mM of H_2_O_2_ for 1 week, mimicking the situation where the device is implanted in the human body and hydrogen peroxide is generated by an inflammatory reaction (Fonseca and Barbosa, 2001; Patrick et al., 2011). The pHEMA encapsulation showed to withstand the test without visible erosion or cracks. An image of the same pHEMA-encapsulated PEDOT-PSS-CNT probe before and after the immersion test is shown in Figures 4A,B.

**Figure 5:**
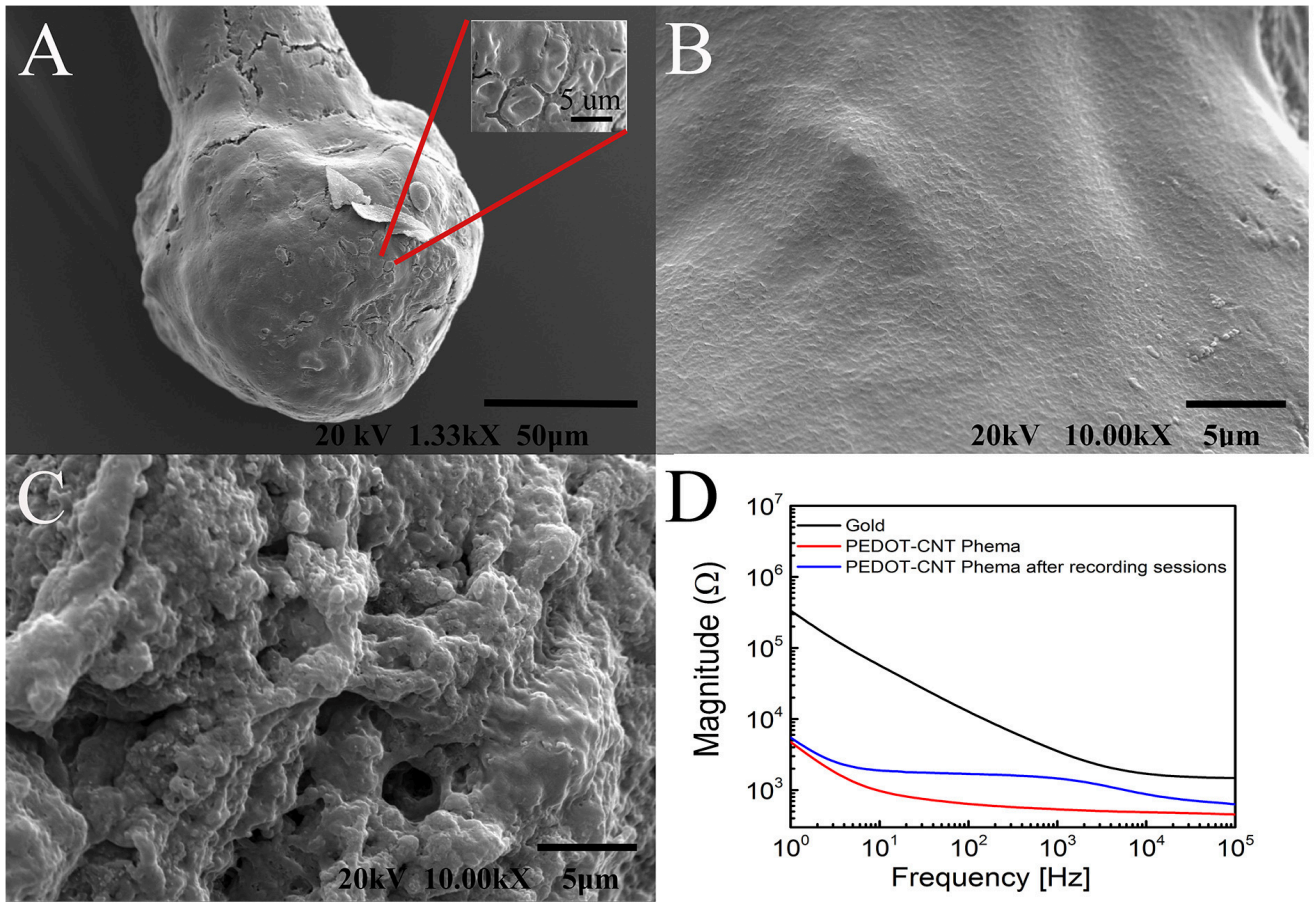
**Representative SEM images of (A) a pHEMA encapsulated PEDOT-PSS-CNT microsphere after recordings in rat and (B) the higher magnification of the pHEMA surface after recordings in rat. (C)** Magnification of the surface of a PEDOT-PSS-CNT coated microsphere (without pHEMA) after recordings in rat. **(D)** Example of magnitude impedance of a pHEMA encapsulated PEDOT-PSS-CNT coated microsphere before and after various recording sessions.

To evaluate the ability of the pHEMA-encapsulated PEDOT-PSS-CNT microspheres to withstand the mechanical stress of brain implants, we investigated their morphology, surface composition and electrochemical properties after implanting them in the cerebral cortex of anesthetized animals to record the neural activity. Figures 5A,B shows an example of SEM image of the pHEMA-encapsulated PEDOT-PSS-CNT microsphere that was previously implanted in rat cortex. One can notice that the main visible consequence is the mere accumulation of tissue debris and erythrocytes on the hydrogel surface that are easily recognizable in the inset of Figure 5A. We have acquired SEM images at the same magnification of the hydrogel surface (Figure 5B) and of a non–encapsulated PEDOT-PSS-CNT microsphere (Figure 5C). Non–encapsulated PEDOT-PSS-CNT microsphere maintains, after the insertion and recording in rat brain, the typically rough and porous surface of the non-coated probe. Conversely, the pHEMA-encapsulated PEDOT-PSS-CNT microsphere keeps the smooth pHEMA surface morphology both before (Figure 3B) and after (Figure 5B) implant and recording. In order to verify that indeed pHEMA is still present after probe implant, we have performed EDS analysis of pHEMA-encapsulated microspheres prior and after implants, comparing results with spectra obtained from non-encapsulated PEDOT-PSS-CNT microspheres and from a reference sample of pHEMA. As we are dealing with a low density material—the hydrogel and the conductive polymer—made from low atomic number elements, one has to take into account that the penetration depth of the electron beam is several micrometers and can reach the underlying microsphere surface. Differences in detected composition between the pristine PEDOT-PSS-CNT devices and the pHEMA encapsulated ones regard mainly the relative amount of detected carbon, gold and sulfur. In the case of pristine PEDOT-PSS-CNT microspheres, one finds a significant sulfur signal that arises from PEDOT-PSS and also a high gold signal from the underlying gold-ball. A smaller sodium signal can also be attributed to PEDOT-PSS. When encapsulated with a pHEMA layer (dry thickness 6.49 ± 2.62 μm, average of 10 samples), gold and sulfur percentage significantly decrease due to the presence of the hydrogel layer that reduces the number of electrons reaching the underlying materials, while the oxygen and carbon signals have values similar to those of pure pHEMA (see Supplementary Table 1S and Supplementary Figure 2S for examples of such spectra). We have then performed EDS analysis on non-encapsulated PEDOT-PSS-CNT microspheres and pHEMA-encapsulated probes that have either been inserted three times in the rat cortex (acute experiment) or have been implanted for 28 days, comparing results with the analysis performed on pristine probes (Table 2). Examples of corresponding spectra are shown in Supplementary Figures 3S, 4S. Comparing the EDS analysis results for each kind of microsphere, we find that the characteristic signatures - higher S and smaller C values for PEDOT-PSS-CNT and the opposite for pHEMA - and preserved after implant. We also compared images and EDS spectra for the same pHEMA-encapsulated probes before and after implant, and an example of results are shown in the Supplementary Figure 5S. This is an indirect but convincing proof that both the PEDOT-PSS-CNT coating and the pHEMA layer withstand implant up to 28 days. Finally, Figure 5D reports an example of impedance magnitude spectra of a pHEMA-encapsulated PEDOT-PSS-CNT microsphere before and after several brain insertions and recording sessions, showing that the impedance is maintained well below 2 kΩ for frequencies above 10 Hz.

**Table 2:** **Relative percentage of chemical elements found by EDS on: PEDOT-PSS-CNT non-encapsulated microspheres before and after acute implantation (mean values for 4 probes, 4 spectra for each probe, 3 insertions); pHEMA-encapsulated microspheres before and after acute implant (mean values for 4 probes, 4 spectra for each probe, 3 insertions); a PEDOT-PSS-CNT non-encapsulated microsphere and a pHEMA-encapsulated one (mean values of 4 spectra each) 28 days after implantation**.

	**O (%weight)**	**C (%weight)**	**S (%weight)**	**Na (%weight)**	**Au (%weight)**
PEDOT-PSS-CNT probe before acute implant	77.9 ± 2.8	14.6 ± 3.3	7.2 ± 1.3	0.2 ± 0.2	–
PEDOT-PSS-CNT probe after acute implant	76.7 ± 4.8	14.5 ± 4.6	6.4 ± 1.4	0.20 ± 0.3	1.3 ± 2.7
pHEMA-encapsulated probe before acute implant	67.9 ± 4.8	31.3 ± 5.5	0.7 ± 1.1	–	–
pHEMA-encapsulated probe after acute implant	69.6 ± 5.4	29.1 ± 5.9	0.5 ± 1.2	0.04 ± 0.1	0.6 ± 1.7
PEDOT-PSS-CNT probe after 28 days implant	75.9 ± 6.4	14.5 ± 2.9	3.7 ± 3.4	0.2 ± 0.1	5.2 ± 4.9
pHEMA-encapsulated probe after 28 days implant	69.5 ± 3.2	30.3 ± 3.4	0.2 ± 0.3	–	–

#### Reduced mechanical mismatch between brain and pHEMA encapsulated electrodes

The Young's modulus value of the pHEMA hydrogel, calculated from the initial linear slope of the stress-strain curve, was *E* = 0.091 ± 0.023 MPa. The lower stiffness of the hydrogel coating as compared to the naked metal electrode, together with the fact that the coating makes the radius larger, helps to reduce the elastic mismatch with cerebral tissue and thus reduces the contact pressure upon the tissue itself when and if small displacements of the implanted electrode occur.

The improvement in this direction can be quantified considering the contact between the electrode tip and the cavity as two conforming surfaces (Goodman and Keer, 1965). The pressure-penetration relationship is not trivial, as the exact conformity, i.e., the (small) clearance between cavity and sphere is not known. However, even without precise quantification of the contact pressure, the dependencies on stiffness and sphere size can be expressed as (Liu et al., 2005):

(3) $$\begin{array}{l} p_{0}\propto \frac{E^{*}}{R} \end{array}$$

Where E^*^ is the reduced modulus:

(4) $$\begin{array}{l} \frac{1}{E^{*}}=\frac{1-\nu_{1}^{2}}{E_{1}}+\frac{1-\nu_{2}^{2}}{E_{2}} \end{array}$$

With *E* and ν the Young's modulus and Poisson ratio of either contacting material, identified with subscripts 1 and 2, and *d* the center-to-center displacement.

We simulate according to this model the relative displacement of the implanted electrode to impinge on the surrounding tissue, already in contact. We consider the relative variation of contact pressure arising from modifications of the parameters E^*^ and *R*, for the sake of comparison. We assume also small displacements, so that we treat each material as a bulk body rather than a coated system, and Poisson ratio ν = 0.49 for PHEMA, which is the typical value of hydrogels, due to their very low compressibility. For cerebral tissue, we take the value *E* = 0.01 MPa, ν = 0.45 (Soza et al., 2005).

From Equation (3), it can be seen that contact pressure is proportional to the reduced modulus E^*^ and to the sphere radius to the power of −1. For a given displacement *d*, the relative variation of contact pressure with respect to the uncoated system (gold, *E* = 79 GPa, Poisson ratio ν = 0.42, *R* = 50 μm) is around 20%. This reduced mechanical mismatch between the brain and the pHEMA encapsulated probe is expected to have positive effects by lowering tissue damage.

### Results of *In vivo* recordings

#### Acute recordings

In order to validate the recording capability of pHEMA-encapsulated microspheres, we analyzed and compared the neural signals acquired from the parietal cortex of six rats during acute recording sessions using six non-encapsulated and six pHEMA-encapsulated PEDOT-PSS-CNT microspheres. During each recording session, 35-min recording traces were acquired. To limit the experimental variability, the same cortical region was recorded, identified on the basis of stereotaxic coordinates (see Section Animal Surgery for Signal Recording). To determine the localization of the microelectrodes a thionin staining was adopted and penetration traces of PEDOT-PSS-CNT and pHEMA-encapsulated microspheres were examined (examples of traces are shown in Supplementary Figure 6S). pHEMA encapsulation did not alter the microspheres capability to record action potentials from single units. In fact, the average SNR calculated for pHEMA encapsulated PEDOT-PSS-CNT microspheres (*n* = 6) is not significantly different from the value of SNR calculated for the non-encapsulated PEDOT-PSS-CNT microspheres (*n* = 6) [1.74 ± 0.15 vs. 1.87 ± 0.26; *F*_(1, 13)_ = 0.18; *p* = 0.67 one-way ANOVA].

#### Chronic recordings and effects on pHEMA encapsulation

pHEMA-encapsulated (22 microspheres) and non-encapsulated PEDOT-PSS-CNT (22 microspheres) were chronically implanted in the parietal cortex of 11 rats (see Table 1 for details). Afterwards, *in vivo* impedance spectra and recordings were acquired weekly, up to 28 days, to evaluate the electrochemical properties and functional integrity of the encapsulated microspheres into the brain.

**Figure 6:**
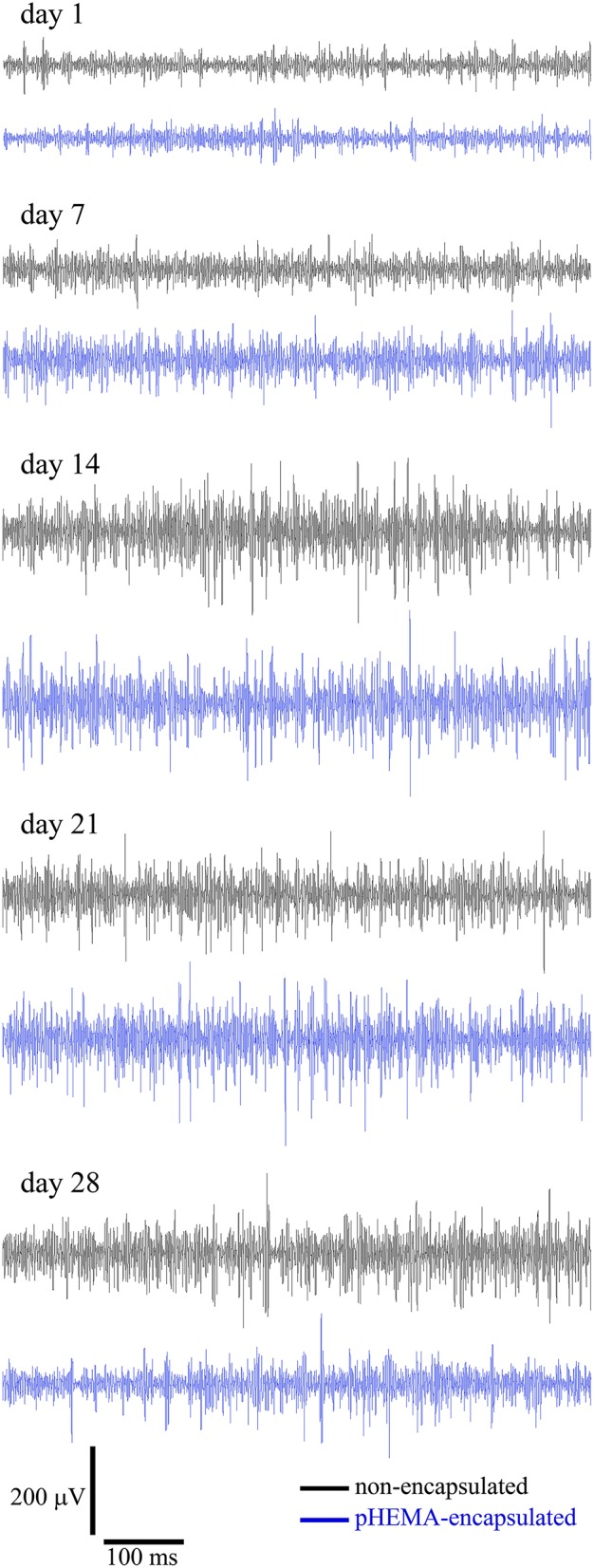
**High-pass filtered (cut-off 250 Hz) traces (800 ms) recorded for non-encapsulated PEDOT-PSS-CNT (black) and pHEMA-encapsulated (blue) microspheres at 1, 7, 14, 21, and 28 days after the implant**.

Figure 6 reports high-pass filtered (cut-off 250 Hz) example traces of 800 ms activity recorded in chronic experiments with non-encapsulated PEDOT-PSS-CNT (black) and pHEMA-encapsulated (blue) microspheres at 1, 7, 14, 21, and 28 days after the implant. In both cases, after the first day post-implant when the spike amplitude was lower, spiking activity remained relatively constant from 7 to 28 days after implant. To verify the spikes recording capability we measured the number of spikes per minute in a range of 1000 s of acquired high-pass filtered data. The results are reported in Table 3.

**Table 3:** **Spikes/min (average ± SD) recorded from 3 animals (4 hemispheres) at 1, 7, 14, 21, and 28 days after implant, and from 6 animals (12 hemispheres) at 1, 7, and 14 days after implant**.

**Days after implant**	**non-encapsulated microspheres**	**pHEMA encapsulated microspheres**
**28 days implants (3 animals, 4 hemispheres)**		
1	1519.0 ± 523.0	1594.2 ± 467.7
7	1504.5 ± 595.7	1635.0 ± 529.7
14	2492.0 ± 139.6	1863.0 ± 786.7
21	1654.3 ± 1151.4	1313.8 ± 505.4
28	1729.8 ± 948.1	1902.4 ± 697.9
**14 days implants (6 animals, 12 hemispheres)**		
1	1133.0 ± 542.8	1259.0 ± 836.1
7	1189.5 ± 712.0	1249.6 ± 903.7
14	1464.2 ± 879.4	1539.3 ± 867.6

*Data of non-encapsulated PEDOT-PSS-CNT and pHEMA-encapsulated PEDOT-PSS-CNT microspheres are shown*.

**Figure 7:**
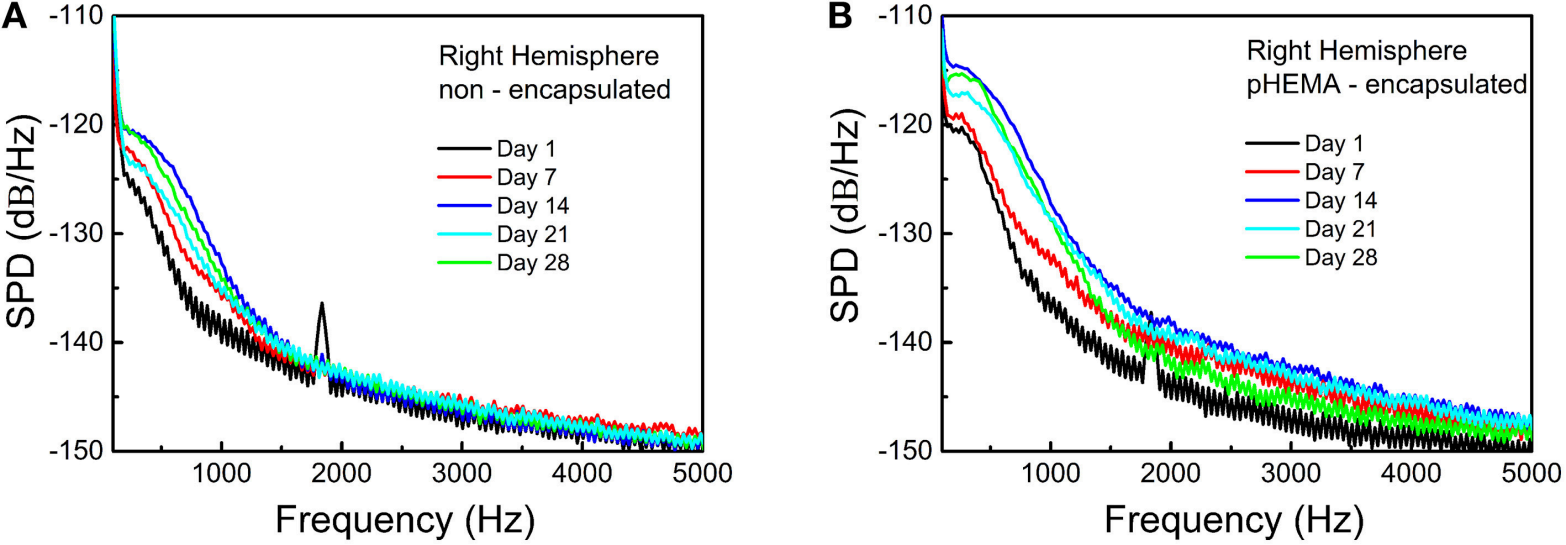
**SPDs obtained for the non-filtered spontaneous activity recorded from the rat brain using non-encapsulated PEDOT-PSS-CNT (A) and pHEMA-encapsulated (B) microspheres at 1, 7, 14, 21, and 28 days after the implant**.

The same results are confirmed by the SPDs (Figure 7) obtained for 10 seconds of the non-filtered spontaneous activity recorded from the same animal using PEDOT-PSS- non-encapsulated CNT microspheres (Figure 7A) and pHEMA-encapsulated (Figure 7B) microspheres at 1, 7, 14, 21, and 28 days after the implant. An example of signal power values over the spike frequency range, computed as the integral of the SPDs of the signals between 250 and 3000 Hz -the frequency range where spikes of individual neurons can be detected- is reported in Supplementary Table 2S. After 28 days both encapsulated and non-encapsulated electrodes were still able to record high-quality spiking activity.

**Figure 8:**
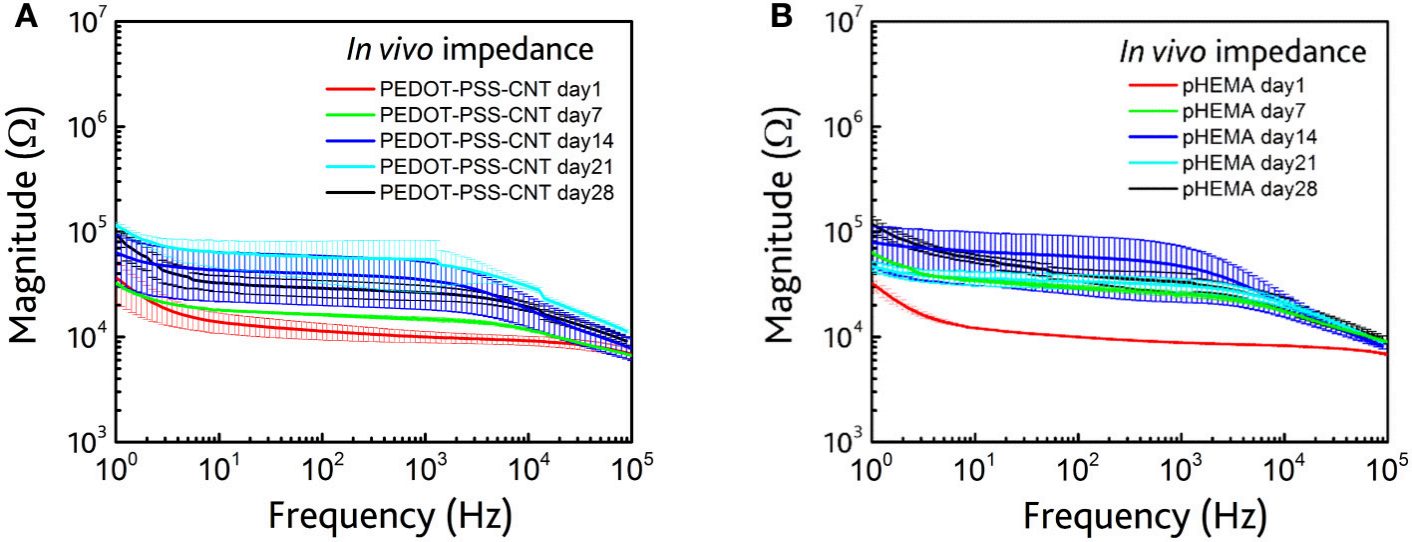
**(A)** *In vivo* impedance spectra (magnitude) of non-encapsulated microspheres at 1 (red), 7 (green), 14 (blue), 21(cyan), and 28 (black) days AFTER the implant. **(B)** *In vivo* impedance spectra (magnitude) of pHEMA encapsulated PEDOT-PSS-CNT coated microspheres at 1 (red), 7 (green), 14 (blue), 21(cyan), and 28 (black) days after the implant.

The impedance spectra behavior in the whole frequency range (1 ÷ 10^5^ Hz), reported in Table 4, is similar for both types of microelectrodes, with an increase of impedance after the first day from the implant. The impedance spectra respectively of non-encapsulated and pHEMA-encapsulated PEDOT-PSS-CNT 1, 7, 14, 21, and 28 days microspheres from the implant are shown in Figure 8. These impedance values stay within the impedance range that allows to efficiently record action potentials during the whole implant period, as previously shown (Figures 6, 7). The implanted microspheres, after being removed from the brain, were analyzed using SEM. This confirmed the persistence of an intact pHEMA encapsulation and no insulation delamination. Figures 12A,B shows some examples of high-magnification SEM images of the surface for non-encapsulated and pHEMA encapsulated PEDOT-PSS-CNT microspheres, respectively, after 28 days from the implant. The PEDOT-PSS-CNT exhibits its typical rough and porous morphology, while the pHEMA encapsulation maintains its smooth surface morphology. When EDS analysis (see Section Sturdiness and Electrochemical Stability of pHEMA Encapsulation and Table 2) was performed on these samples, we found results similar to those obtained on pristine samples, indicating that the pHEMA encapsulation withstands brain insertion and 28 days implant.

**Table 4:** **Impedance values at 1 kHz of pHEMA-encapsulated and non-encapsulated PEDOT-PSS-CNT coated microspheres**.

**Days after the implant**	**PEDOT-PSS-CNT**	**pHEMA encapsulated**
1	9.9 ± 1.2 kΩ	8.8 ± 0.2 kΩ
7	14.7 ± 0.6 kΩ	25.6 ± 1.6 kΩ
14	34.8 ± 16.8 kΩ	46.9 ± 25.7 kΩ
21	54.6 ± 28.2 kΩ	30.8 ± 4.8 kΩ
28	26.3 ± 4.2 kΩ	33.2 ± 7.1 kΩ

#### Immunofluorescence

Immunofluorescence analysis was performed on four non-encapsulated and four pHEMA-encapsulated PEDOT-PSS-CNT microspheres tracks of three hemispheres at 2 weeks and 4 weeks after the implant. The density of GFAP immunoreactivities surrounding the microsphere tracks was measured with ImageJ software on images at 8-bit color of 4 tracks acquired using a 20 × lens. The quantification box that delimited the region of interest was chosen considering the extension of the reactive glia near the track and the same box in the same slice was used to measure the background values captured distant from the track. The density values of background was subtracted from the density of GFAP fluorescence.

**Figure 9:**
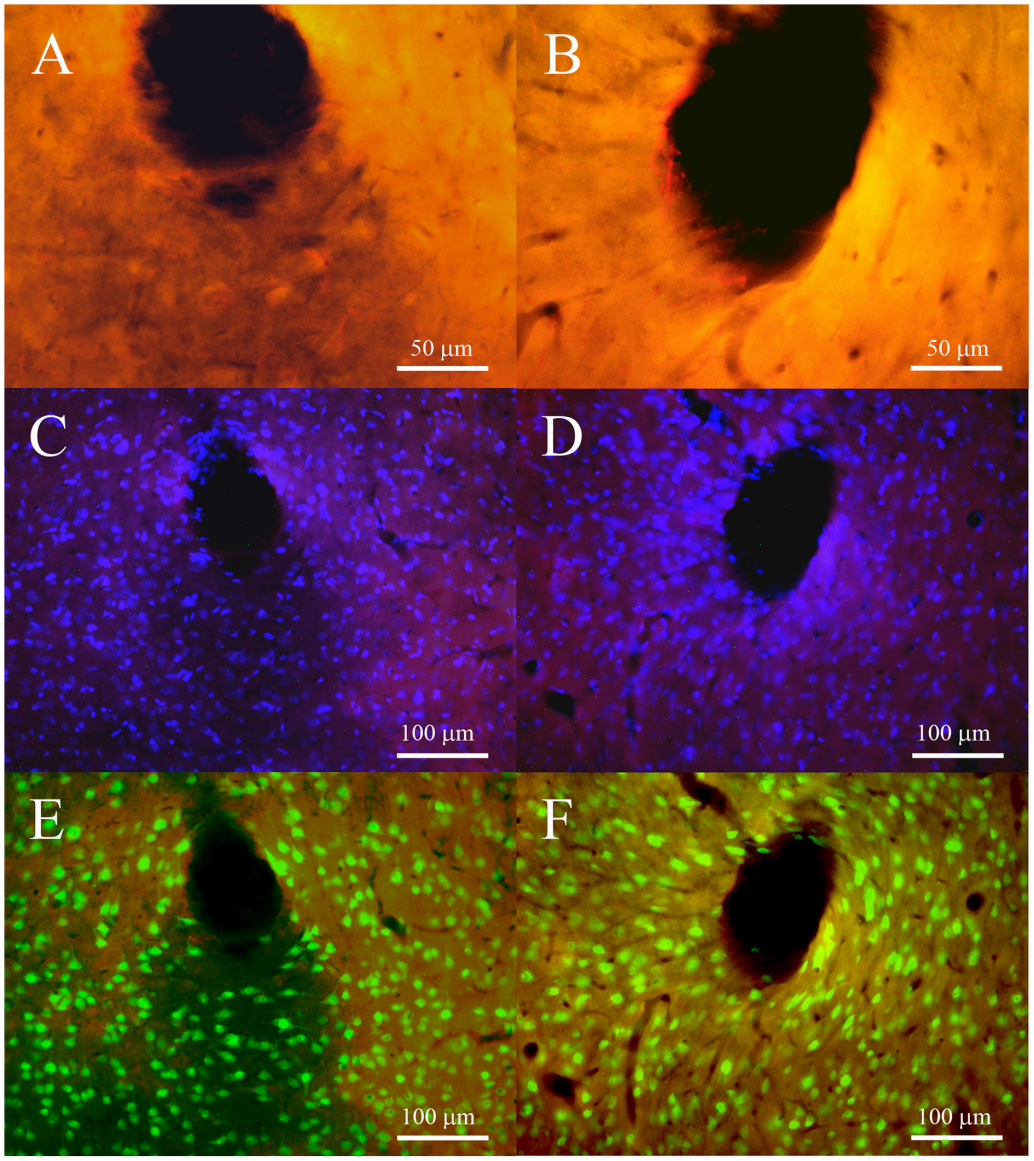
**Fluorescence microscopy images of tracks for non-encapsulated (left panels) and pHEMA-encapsulated (right panels) microspheres at 2 weeks after implant. (A,B)** show the GFAP-positive cells (red) at 40 × to underline the morphology of astrocytes; **(C,D)** cell nuclei (blue) merged on GFAP staining to show the density of DAPI surrounding tracks; **(E,F)** neuronal nuclei (green) merged with GFAP staining to show the presence of neuronal bodies surrounding the track.

**Figure 10:**
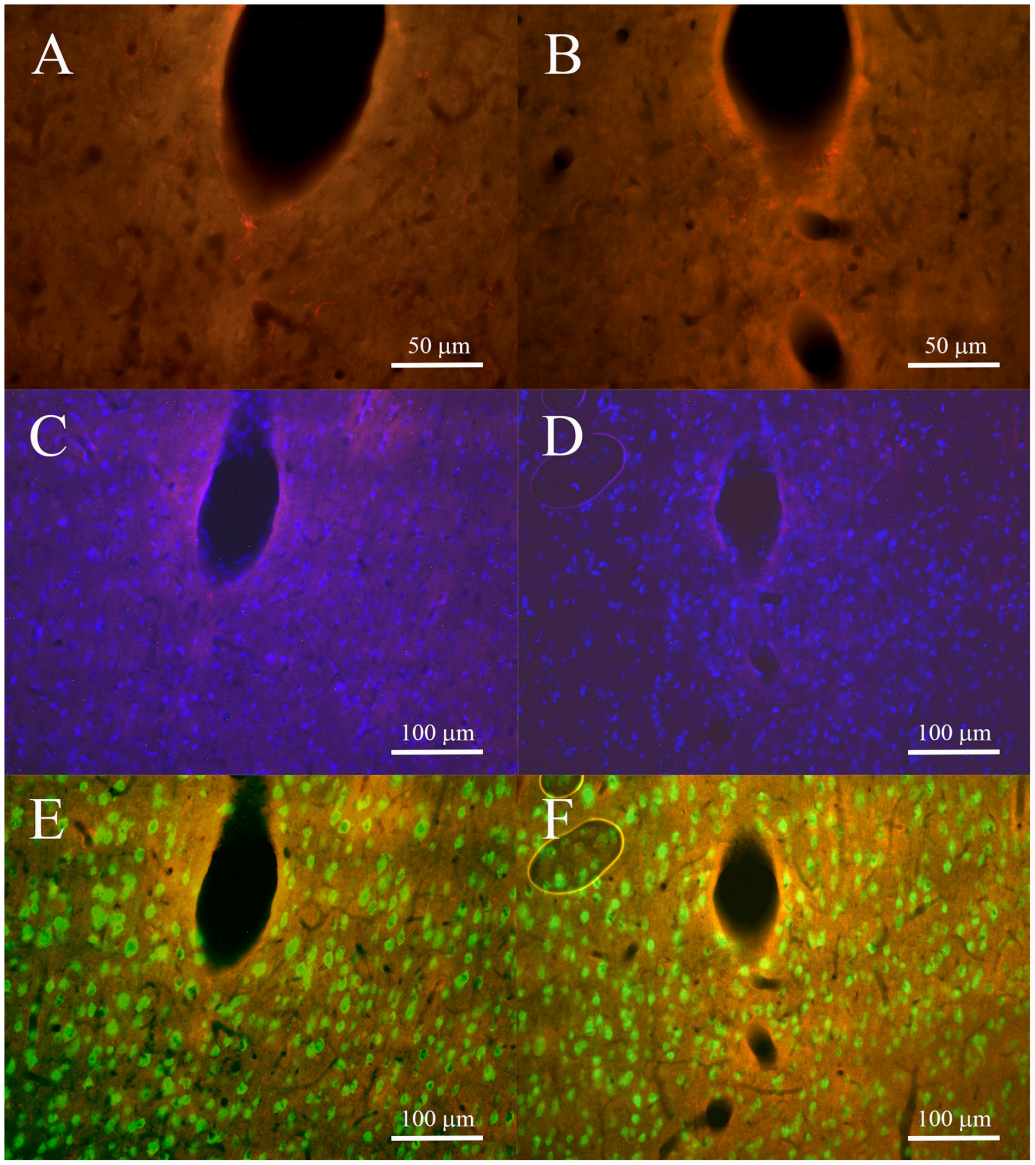
**Fluorescence microscopy images of tracks for non-encapsulated (left panels) and pHEMA-encapsulated (right panels) microspheres 4 weeks after implant. (A,B)** show the GFAP-positive cells (red) at 40 × to underline the morphology of astrocytes; **(C,D)** cell nuclei (blue) superimposed on GFAP staining to verify the density of DAPI surrounding the tracks; **(E,F)** neuron nuclei (green) superimposed on GFAP to show the presence of neuronal bodies surrounding the tracks.

In the 2-weeks implanted animals the levels of GFAP for both non-encapsulated and pHEMA encapsulated PEDOT-PSS-CNT microspheres (Figures 9A,B) were similar, with 43.84% and 42.91% of intensity compared to the background, respectively. The activated astrocytes presented the same extension in surrounding tracks for both type of microelectrodes (28–135 μm for non-encapsulated and 29–141 μm encapsulated ones). After 4 weeks of implant GFAP expression (Figures 10A,B) increased by 28.95% with respect to the background with non-encapsulated microspheres and by 37.69% with pHEMA-encapsulated ones. GFAP staining surrounding the tracks was 15–150 μm for non-encapsulated and 10–100 μm for pHEMA-encapsulated microspheres.

**Figure 11:**
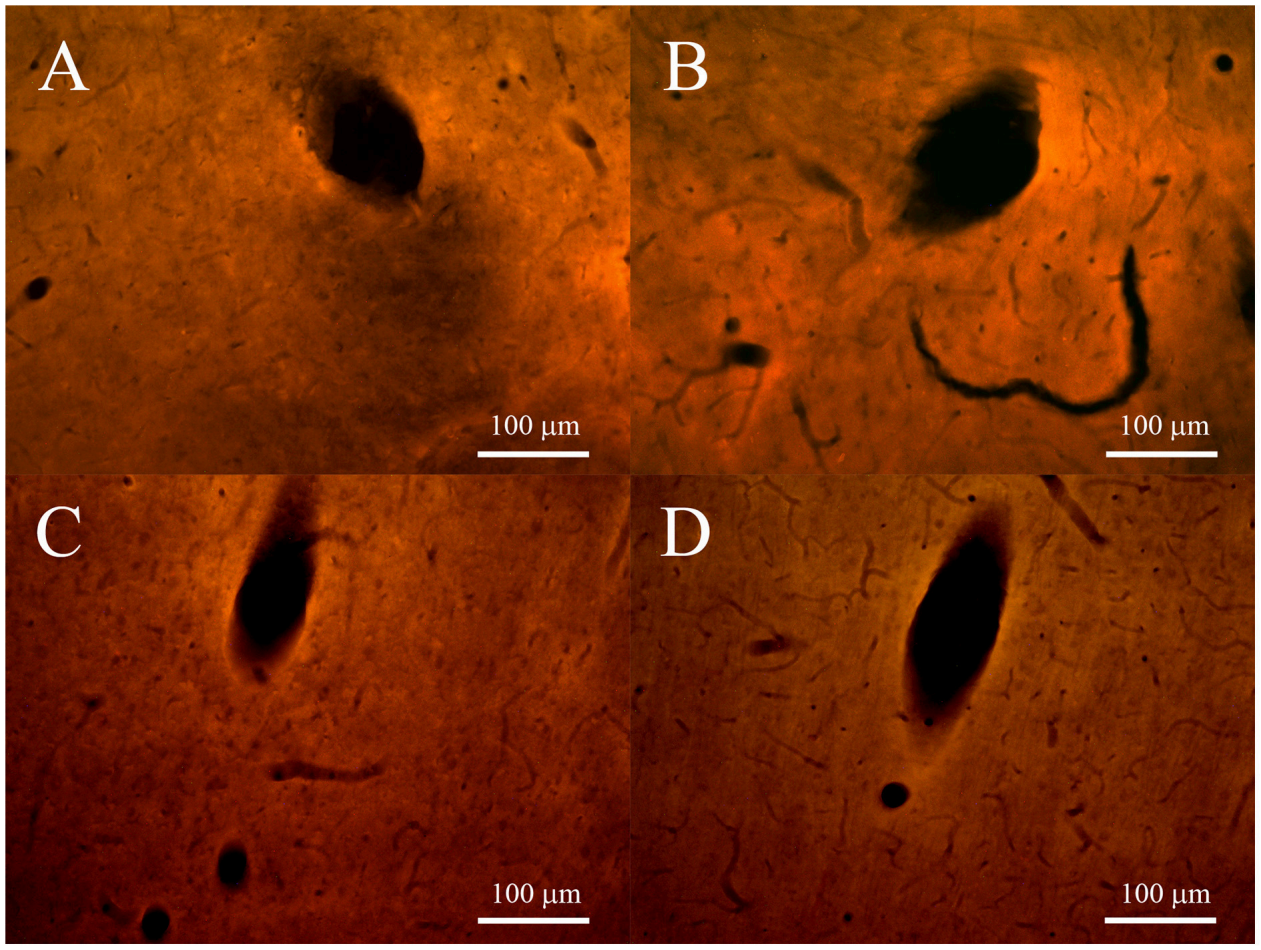
**Fluorescence microscopy images of tracks for non-encapsulated (left panels) and pHEMA-encapsulated (right panels) microspheres, 2 and 4 weeks after the implant. (A,B)** show the ED1-positive cells (red) at 20 × at 2 weeks; **(C,D)** show the ED1-positive cells (red) at 20 × at 4 weeks.

As shown in Figures 9, 10, at both 2 and 4 post-implant weeks and for both types of microelectrode, the number of GFAP stained cell nuclei surrounding the tracks was increased (Figures 9C,D, 10C,D), but without any detectable neuronal loss (Figures 9E,F, 10E,F). Few ED1-positive cells were observed at 2 post-implant weeks in proximity of tracks for both type of microelectrodes (Figures 11A,B), while no evidence of ED1-positive cells was identified at 4 weeks (Figures 11C,D).

**Figure 12:**
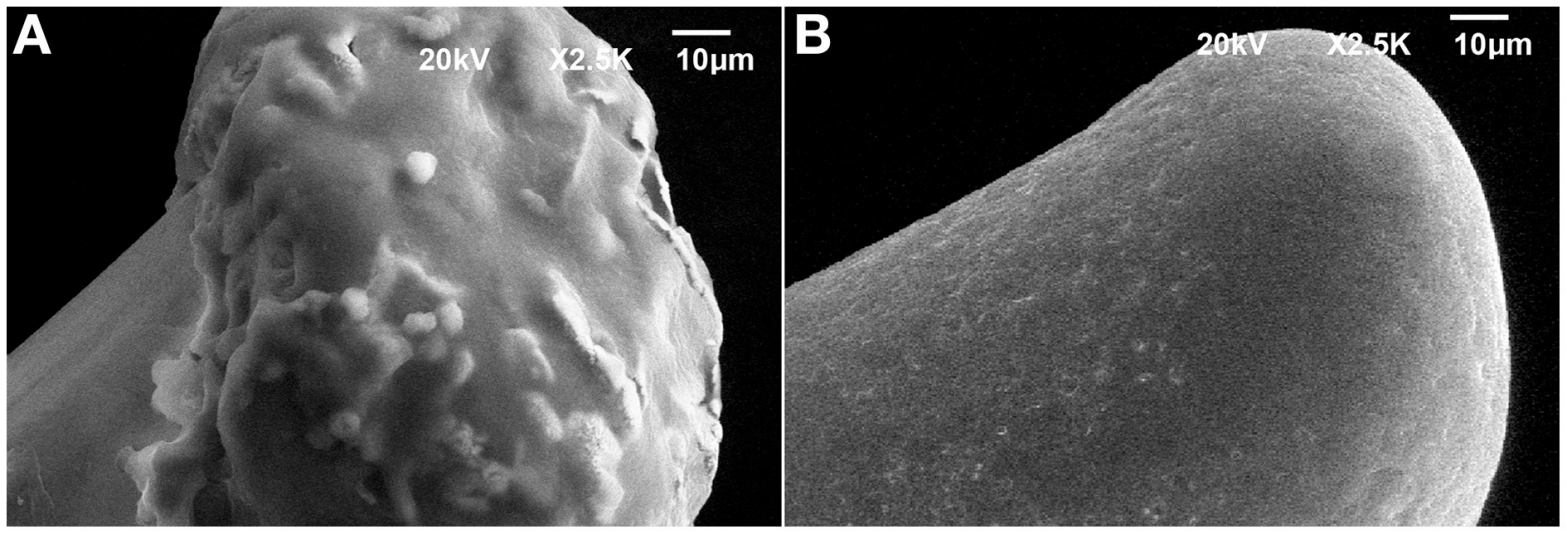
**Representative SEM images of (A) PEDOT-PSS-CNT coated microsphere (without pHEMA) and (B) pHEMA encapsulated PEDOT-PSS-CNT coated microsphere after 28 days of permanence in rat brain**.

## Discussion

The aim of this paper was to combine in a single device several techniques for improving long-term reliability of neural interfaces and stability of recordings. These techniques have been: (1) the use of gold nanostructured microspheres electrochemically grown at the free end of a thin tether; (2) the use of high surface area PEDOT-PSS-CNT coatings; (3) the encapsulation of the PEDOT-PSS-CNT coated microspheres with a soft, synthetic and permanent biocompatible hydrogel.

The starting point of this work was the systematic use of the device, presented in our previous studies (Castagnola et al., 2014b, 2015), made from an electrochemically grown gold sphere (the recording site) at the free end of a thin insulated platinum wire. We have here used 50 μm diameter Pt wires with stiffness of 0.63 N/mm, much thinner and flexible when compared to a commercial probe with typical stiffness of 13 N/mm (TREC microelectrodes by Thomas Recording, Germany), and it is possible to extend the results using the same gold spheres grown at the end of much thinner wires (e.g., 12 μm diameter platinum core, Castagnola et al., 2015) thus taking full advantage of the high flexibility of the tether. Our device is able to better accommodate motions of the brain with respect to the skull and, at the same time, has a recording surface sufficient in size to allow electrochemical properties adequate for both recording and stimulation experiments. We have shown that it is possible to reduce the impedance and increase the charge transfer capability of these microprobes by coating them with a PEDOT-PSS-CNT high surface area composite (Castagnola et al., 2015). For this study we produced a very large number of PEDOT-PSS-CNT coated microspheres, consistently reproducing previous results for improvement of impedance and charge transfer capability. The differences in electrical behavior between the microstuctured gold and the PEDOT-PSS-CNT composite, can be attributed to two contributions: one coming from the PEDOT:PSS conduction mechanism and the other from the nanoporous CNT scaffold. In fact, compared to gold, the conducting polymer (PEDOT:PSS) is able to conduct both ionic and electronic current, enhancing the efficiency of signal transduction (Berggren and Richter-Dahlfors, 2007; Abidian and Martin, 2008; Rivnay et al., 2014). The high capacitance of the material originates from the pseudo-or redox-capacitance of PEDOT (Bard and Faulkner, 2001; Gerwig et al., 2012). Charge transfer capacitance is then further enhanced by the nanometer scale porosity of the CNT scaffold, that increases the surface area of PEDOT:PSS available to the solution (Gerwig et al., 2012; Samba et al., 2014). In fact, PEDOT-PSS-CNT is a very promising material for neural interfaces as, with respect to conventional metal electrodes, shows higher conductivity, better electrochemical stability, greater mechanical properties and has shown to perform well during *in vivo* recordings (Gerwig et al., 2012; Castagnola et al., 2013; Chen et al., 2013; Kozai et al., 2015; Samba et al., 2015).

The presence of CNTs or other nanomaterials onto the microelectrodes surface, often rises concerns regarding possible health hazards, especially when considering a clinical application of the devices (Oberdörster et al., 2005; Resnik and Tinkle, 2007; Sahoo et al., 2007). To prevent this risk, we previously demonstrated that a fibrin hydrogel encapsulation of high-density arrays of epicortical microelectrodes is electrically transparent and can provide a mechanically/chemically stable barrier that avoids direct exposure of the brain to nanomaterials while maintaining all the electrical advantages deriving from the nanostructured electrode surface (Castagnola et al., 2013, 2014b). However, the fibrin coating of intracortical microelectrodes implanted in rat brain is almost completely reabsorbed in a few weeks *in vivo* (De Faveri et al., 2014), making it eligible only as a barrier for acute implants. As our aim was to improve the quality of long-term electrode implants, we have introduced here the use of the synthetic hydrogel pHEMA. In general, compared to hydrogels composed of only natural materials, synthetic hydrogels allow obtaining a rigorous control of their polymerization, degradation, and biocompatibility. Furthermore, they are more chemically defined and biologically inert than those based on natural materials, decreasing the possibility of immunorejection when implanted into the brain (Aurand et al., 2012). We have focused our attention on the pHEMA hydrogel, as it is a widely studied polymer that has found several biomedical applications, such as contact lenses, bioadhesive gels for drug delivery applications, and thrombo-and fibro-resistant coatings (Mohoned et al., 2005).

The first essential result has been the demonstration that pHEMA encapsulation of the PEDOT-PSS-CNT coated microspheres fully preserves the superior electrochemical performance of high surface areas, while introducing a physical barrier that reduces the hypothetical risk of a direct contact of the tissue with the nanomaterials. Moreover, mechanical measurements have shown that the pHEMA hydrogel coating reduces by 20% the contact pressure between electrode and brain tissue, a key factor for the reduction of foreign body rejection (Polikov et al., 2005; Lind et al., 2010; Aregueta-Robles et al., 2014; Agorelius et al., 2015; Kozai et al., 2015). We have also shown that pHEMA-encapsulated microelectrodes were suitable for *in vivo* recordings in the rat brain, preserving the recording quality of the PEDOT-PSS-CNT microspheres without pHEMA. To validate this result, it was essential to find out whether the pHEMA encapsulation stays in place both during acute and chronic implants. We found that pHEMA withstands the mechanical stress occurring during several consecutive brain insertions in acute experiments. Concerning the ability to endure the chemical stress due to the tissue reaction to implant, a first encouraging result was the absence of degradation or crack formation on the pHEMA coated microspheres after their immersion of in a phosphate buffered saline (PBS) solution containing 30mM of H_2_O_2_ for 1 week, mimicking the situation where the device is implanted in the human body and hydrogen peroxide is generated by an inflammatory reaction. The examination of pHEMA-coated gold microspheres implanted for 1 month in the rat cortex, finally showed that the hydrogel is not degraded by the permanence in the brain and is not metabolized as the fibrin hydrogel used in previous studies (De Faveri et al., 2014).

Once established that the pHEMA is a viable solution, we have investigated the quality of recordings by directly comparing results in acute experiments on rats obtained with non-encapsulated PEDOT-PSS-CNT coated microspheres. The absence of significant differences in average SNR ratio between non-encapsulated and encapsulated PEDOT-PSS-CNT microspheres proves that the pHEMA hydrogel is suitable for avoiding the contact of nanomaterials with the brain tissue without significant adverse effects on the electrode performance.

The next step was the validation of the pHEMA encapsulation in chronic implants. The comparative behavior of pHEMA encapsulated and non-encapsulated PEDOT-PSS-CNT microspheres implanted in the same animal and characterized by acquiring *in vivo* impedance spectra shows a similar trend after 1, 7, 14, 21, and 28 days. During the same time interval, both electrodes efficiently maintained their ability to capture action potentials. Recent results have shown that PEDOT-PSS-CNT coatings improve chronic spike recording stability (Kozai et al., 2015) and our preliminary data indicates that the pHEMA encapsulation does not interfere with these PEDOT-PSS-CNT properties and stably insulates the nanomaterial from the tissue even after a prolonged implant.

Immunofluorescence revealed that implant of the microsphere microelectrodes produces at 2 and 4 weeks a contained glial response. In particular, the activated astrocytes present an extension of their processes smaller than 150 μm around the electrode track and a more compact mesh in the proximity of the track thick about 20 μm, also pointed out by an increase of cell nuclei revealed by DAPI staining. At 4 weeks after implant the pHEMA encapsulation produces an increase of glia response, as shown by GFAP expression. Moreover, after implant, only a few activated microglia, the other major glial cells involved in the inflammatory response, are evident at 2 weeks in the proximity of the tracks. These limited manifestations of inflammatory process are not paralleled by neuron loss around the tracks, as revealed by the presence of nuclear fluorescence and testified by the capability of both type of electrodes to record neural activity up to 28 days after the implant.

Overall, our results indicate that the goal of shielding from the tissue the PEDOT-PSS-CNT nanomaterial to minimize the possibility that detached CNTs are shed from the coating inside the tissue is fully met by the pHEMA encapsulation, thus bringing the use of the high performance nanocomposite one step closer to clinical applications.

## Author contributions

EC produced and characterized gold microspheres, PEDOT-CNT coatings, pHEMA encapsulation and substantially contributed to the manuscript writing, EM designed and performed neural recording experiments, histology, performed immunocytochemical analysis and contributed to neural recording analysis, SD contributed to neural implants and recording analysis, FC and EZ performed immunocytochemical analysis, LC performed mechanical characterization of pHEMA hydrogel, LF designed and supervised the project, revised the paper, DR supervised experiments and wrote the paper.

### Conflict of interest statement

The authors declare that the research was conducted in the absence of any commercial or financial relationships that could be construed as a potential conflict of interest.
