# Supplementary material for: pHEMA Encapsulated PEDOT-PSS-CNT Microsphere Microelectrodes for Recording Single Unit Activity in the Brain
Source: Front Neurosci. 2016 Apr 18;10:151. doi: 10.3389/fnins.2016.00151 (PMC4834343; doi:10.3389/fnins.2016.00151)
Supplement: Supplementary file 1 [file DataSheet1.docx]

Supplementary Material

pHEMA encapsulated PEDOT-PSS-CNT microsphere microelectrodes for recording single unit activity in the brain

Elisa Castagnola, Emma Maggiolini, Luca Ceseracciu, Francesca Ciarpella, Elena Zucchini, Sara De Faveri, Luciano Fadiga, Davide Ricci*

*** Correspondence:** Corresponding Author: davide.ricci@iit.it


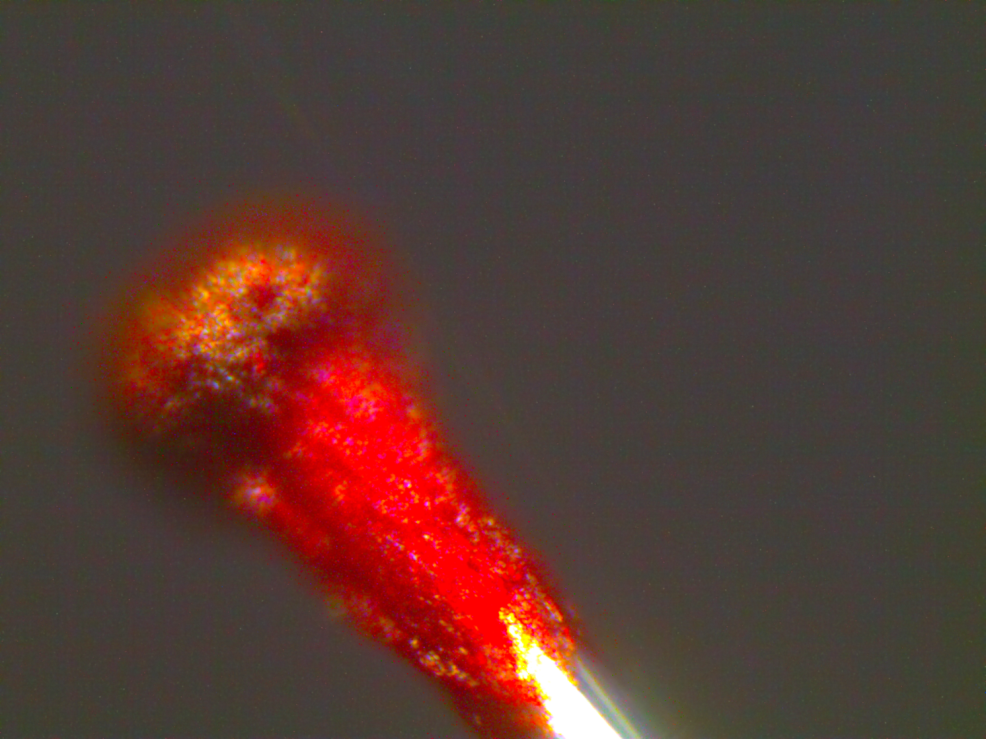


**Supplementary Figure 1S.** Representative optical image of a gold microsphere after encapsulation in dyed pHEMA using a precursor solution containing Ponceau Xylidine dye (5mg/ml).


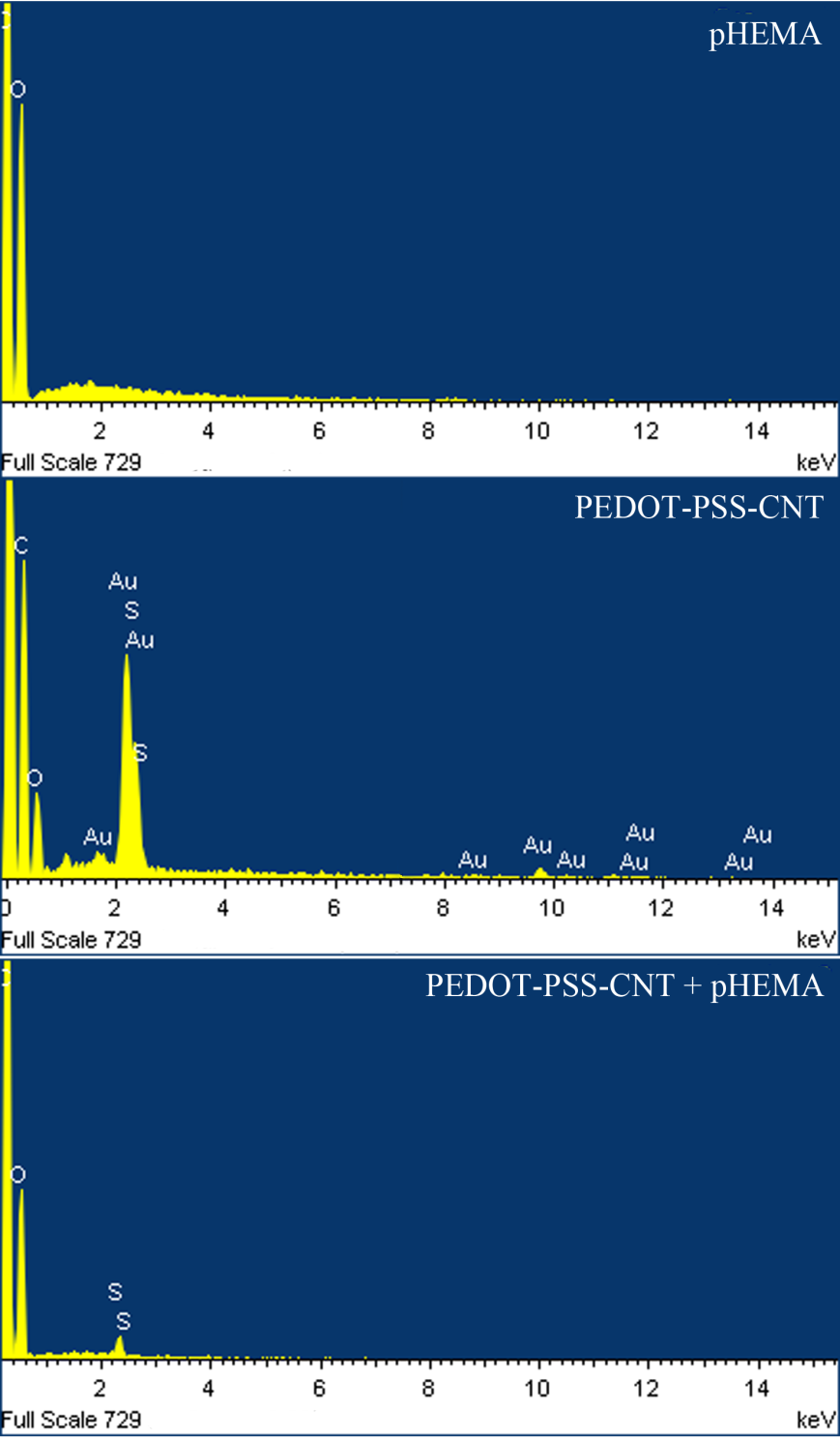


**C**

**B**

**A**

**Supplementary Figure 2S.** Representative spectra obtained by EDS on a pHEMA bulk sample (A), on a PEDOT-PSS-CNT microsphere (B), on a pHEMA-encapsulated PEDOT-PSS-CNT microsphere (C).


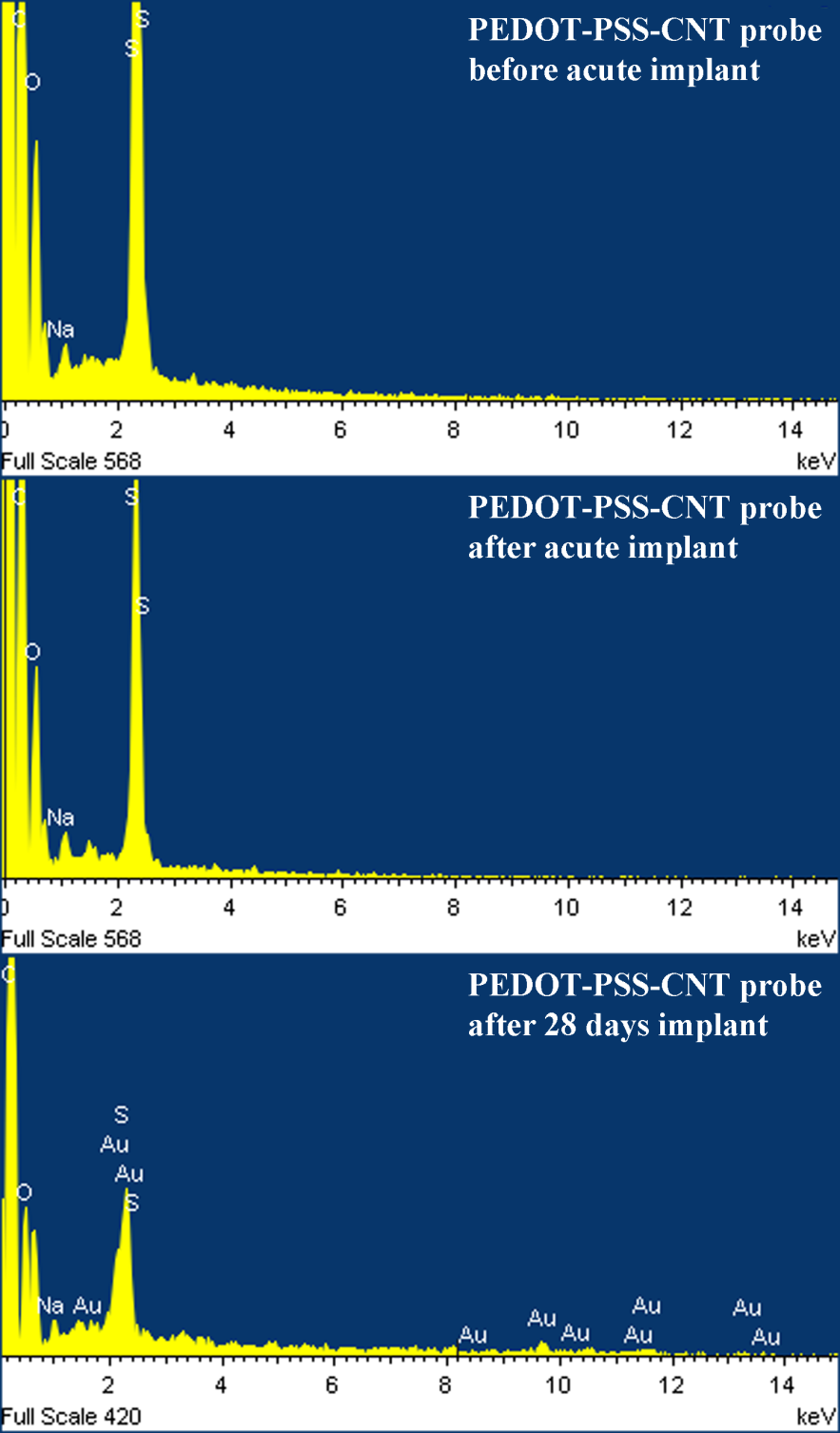


**C**

**B**

**A**

**Supplementary Figure 3S.** Representative spectra obtained by EDS on: a PEDOT-PSS-CNT microsphere before (A) and after (B) acute recordings (3 penetrations); (C) a PEDOT-PSS-CNT microsphere after 28 days implant.

**C**

**B**

**A**


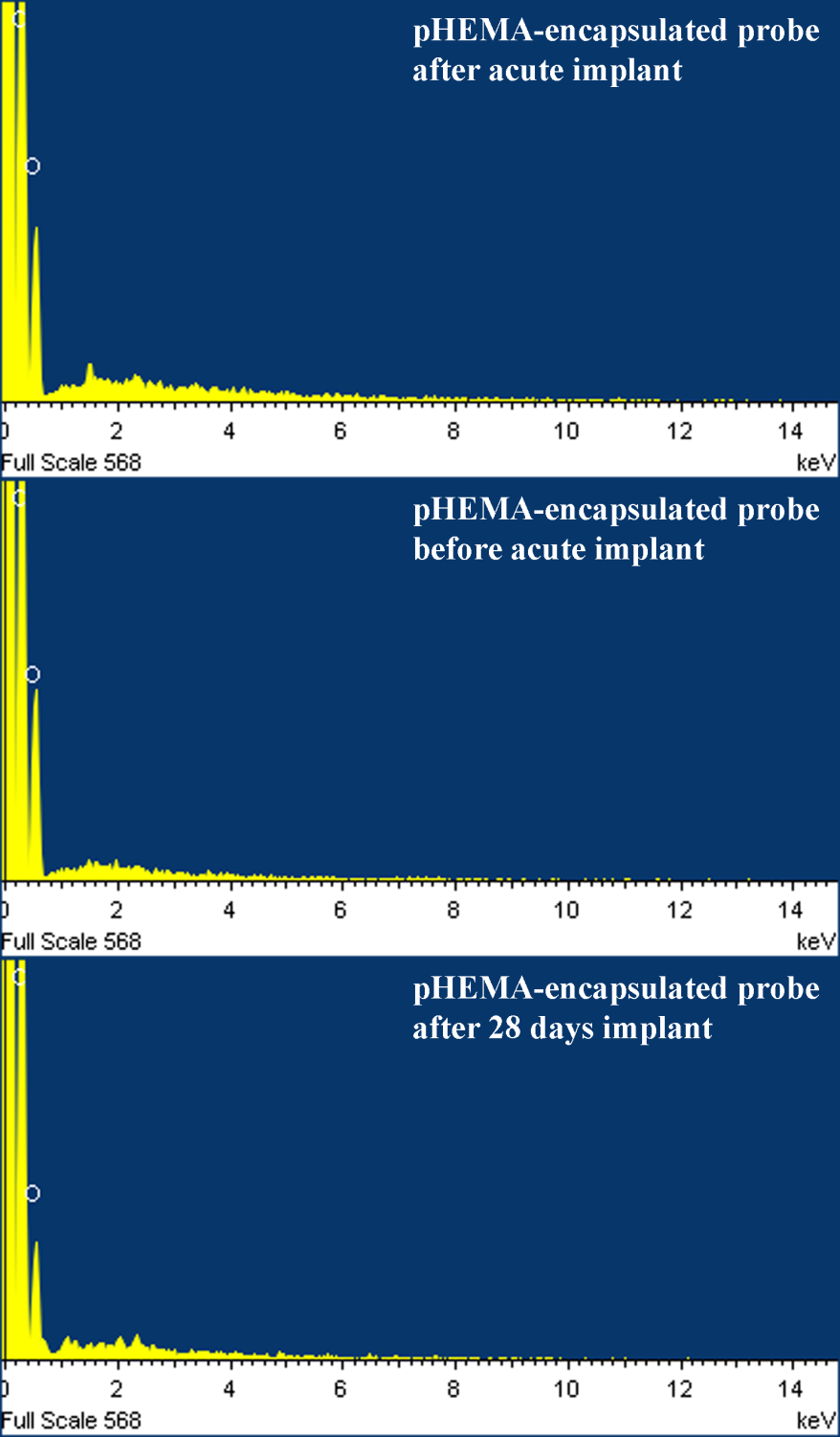


**Supplementary Figure 4S.** Representative spectra obtained by EDS on: a pHEMA-encapsulated microsphere before (A)and after (B) acute recordings (3 penetrations); a pHEMA-encapsulated microsphere after 28 days implant (C).

**Supplementary Figure 5S.** Images and EDS spectra of the same pHEMA encapsulated probe before (A) and after (B) three brain penetrations.


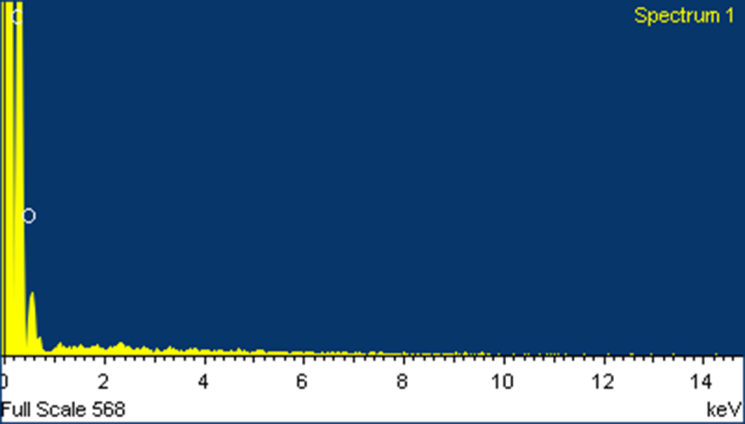

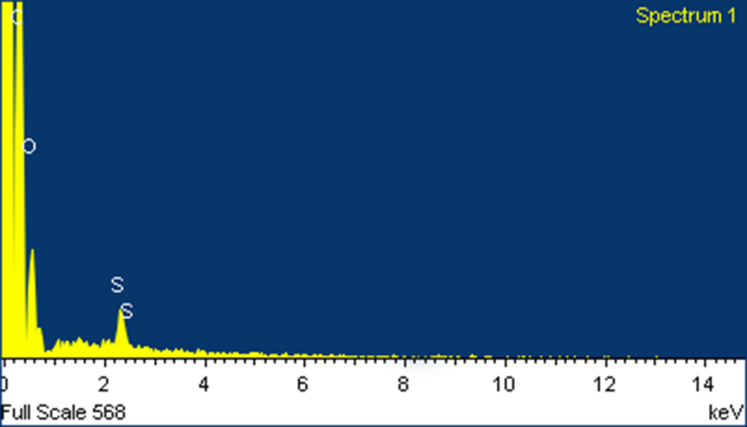

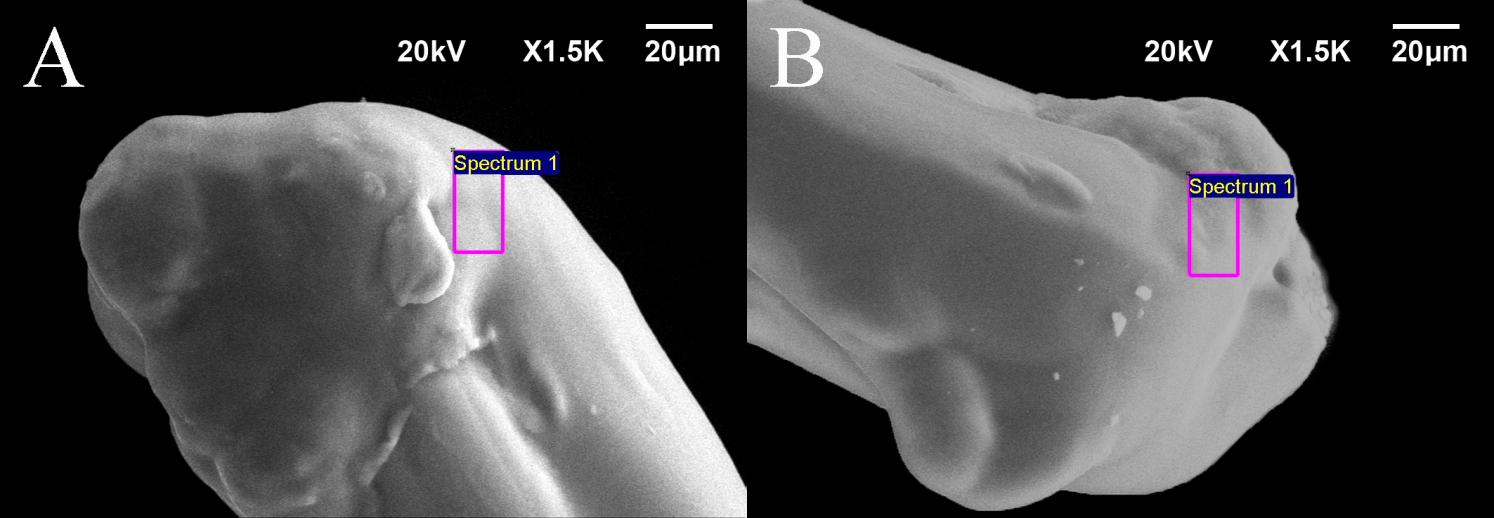

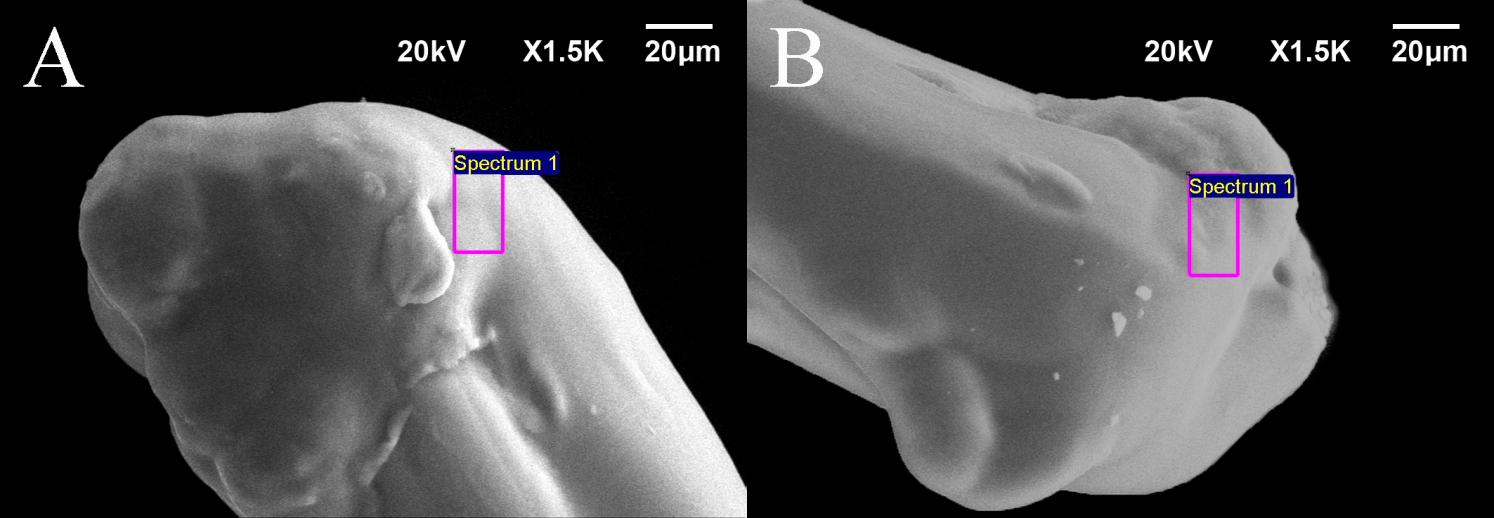


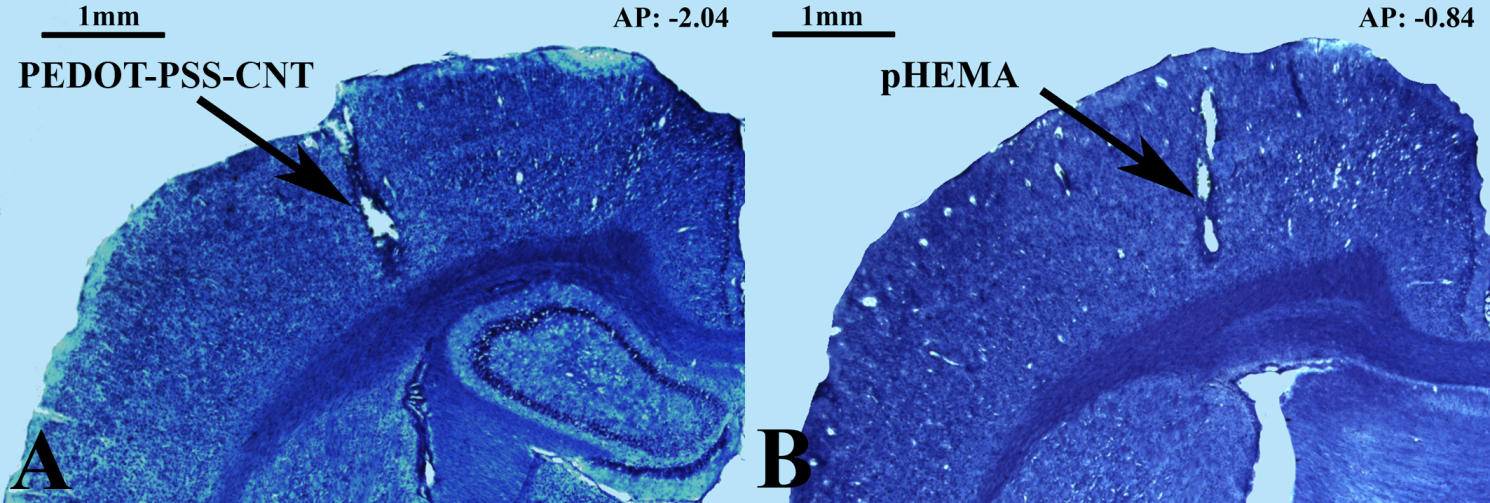


**Supplementary Figure 6S.** Examples of thionin stained sections of rat brain showing microelectrode tracks. Tracks in the rat somatosensory cortex of (A) PEDOT-PSS-CNT coated microelectrode and (B) pHEMA-encapsulated PEDOT-PSS-CNT coated microelectrode. AP, antero-posterior coordinate of the section relative to bregma.

|  | **O**  (%weight) | **C**  (%weight) | **S**  (%weight) | **Na**  (%weight) | **Au**  (%weight) |
| --- | --- | --- | --- | --- | --- |
| **pHEMA reference** | 59.92±1.15 | 40.06±1.19 | -- | -- | -- |
| **PEDOT-PSS-CNT probe** | 61.09±4,36 | 18.80± 1.96 | 3.97±1.12 | 0.32±0.35 | 13.57±6.84 |
| **pHEMA-encapsulated probe** | 62.24±0.75 | 36.24±3.12 | 0,51 ±0,68 | -- | 2.01 ±2.38 |

**Supplementary Table 1S.** Relative percentage of chemical elements found on reference pHEMA sample, PEDOT-PSS-CNT non-encapsulated microspheres (N=2) and pHEMA-encapsulated microspheres (N=5) using EDS.

|  | **Signal Power (dbµV^2^) in 250-3000Hz** | |
| --- | --- | --- |
| **Days** | **pHEMA-encapsulated** | **non-encapsulated** |
| **1** | 1940 | 690 |
| **7** | 2800 | 1300 |
| **14** | 11000 | 3000 |
| **21** | 6800 | 1500 |
| **28** | 8900 | 2400 |

**Supplementary Table 2S.** Example of signal power spectrum values over the spike frequency range, computed as the integral of the SPDs of the signals in the 250-3000 Hz range, the frequency range where spikes of individual neurons can be detected.
